# Identification of 6-(piperazin-1-yl)-1,3,5-triazine as a chemical scaffold with broad anti-schistosomal activities

**DOI:** 10.12688/wellcomeopenres.16069.2

**Published:** 2020-11-13

**Authors:** Gilda Padalino, Iain W. Chalmers, Andrea Brancale, Karl F. Hoffmann

**Affiliations:** 1 Institute of Biological, Environmental and Rural Sciences (IBERS), Aberystwyth University, Aberystwyth, Wales, SY23 3DA, UK; 2 School of Pharmacy and Pharmaceutical Sciences, Cardiff University, Cardiff, Wales, CF10 3NB, UK

**Keywords:** Schistosoma mansoni, Schistosomiasis, histone methylation, SET, MLL, drug discovery

## Abstract

**Background: **Schistosomiasis, caused by infection with blood fluke schistosomes, is a neglected tropical disease of considerable importance in resource-poor communities throughout the developing world. In the absence of an immunoprophylactic vaccine and due to over-reliance on a single chemotherapy (praziquantel), schistosomiasis control is at risk should drug insensitive schistosomes develop. In this context, application of *in silico* virtual screening on validated schistosome targets has proven successful in the identification of novel small molecules with anti-schistosomal activity.

**Methods: **Focusing on the *Schistosoma mansoni* histone methylation machinery, we herein have used RNA interference (RNAi), ELISA-mediated detection of H3K4 methylation, homology modelling and *in silico* virtual screening to identify a small collection of small molecules for anti-schistosomal testing. A combination of low to high-throughput whole organism assays were subsequently used to assess these compounds’ activities on miracidia to sporocyst transformation, schistosomula phenotype/motility metrics and adult worm motility/oviposition readouts.

**Results: **RNAi-mediated knockdown of *smp_138030/smmll-1* (encoding a histone methyltransferase, HMT) in adult worms (~60%) reduced parasite motility and egg production. Moreover, *in silico* docking of compounds into Smp_138030/SmMLL-1’s homology model highlighted competitive substrate pocket inhibitors, some of which demonstrated significant activity on miracidia, schistosomula and adult worm lifecycle stages together with variable effects on HepG2 cells. Particularly, the effect of compounds containing a 6-(piperazin-1-yl)-1,3,5-triazine core on adult schistosomes recapitulated the results of the *smp_138030/smmll-1* RNAi screens.

**Conclusions: **The biological data and the structure-activity relationship presented in this study define the 6-(piperazin-1-yl)-1,3,5-triazine core as a promising starting point in ongoing efforts to develop new urgently needed schistosomicides.

## Introduction

Schistosomiasis is a parasitic disease caused by a blood fluke trematode belonging to the genus *Schistosoma*. The estimated Disability-Adjusted Life Years (DALYs) ^1^ lost for schistosomiasis is 25–28 million and it ranks second only to malaria in terms of public and economic health importance attributable to a human parasitic disease in endemic countries ^2^.

In a scenario where schistosomiasis remains a major cause of morbidity and mortality within developing countries ^2^, the sustainable use of praziquantel as the only currently licensed drug to treat this neglected tropical disease (NTD) remains vulnerable ^3, 4^. Therefore, there is an urgent need to identify novel drugs as an alternative or combinatorial treatment for this disease.

In the search for new anti-schistosomal entities, we and others have chosen to investigate epigenetic processes due to their role in regulating critical aspects of the schistosome life cycle. For example, both cytosine methylation and histone acetylation have previously been shown to control facets of parasite motility, fecundity, developmental progression and survival ^5– 8^. With the Structural Genomics Consortium (SGC) ^9^ making a collection of epigenetic probes/epigenetic inhibitors available for research on NTD-causing pathogens, other histone modifying enzyme (HME) machinery components have recently been identified as new anti-schistosomal targets ^10^. Amongst the SGC collection tested by our laboratory, compounds targeting histone methylation regulators (GSK484, GSK-J4, A-196, MS023, LLY-507, BAY-598, GSK343 and UNC1999) demonstrated potent activities on both schistosomula and adult schistosomes ^10^.

When considering currently known post-translational modifications of histones, methylation is the second most abundant after acetylation ^11, 12^. This epigenetic mark is responsible for chromatin remodelling and subsequent transcription factor accessibility, because methylation alters the interactions between adjacent nucleosomes and the arrangement of the double stranded DNA wrapped around them ^11, 13^. The HMEs involved in this process use the cofactor S-adenosyl-methionine (SAM) to transfer a methyl group to the side chains of specific amino acids on the histone tails (lysine or arginine residues according to the specificity of the histone methyltransferases - HMTs) ^14^. This epigenetic mark is removed by another class of epigenetic enzyme called the histone demethylases (HDMs) ^15^. Recent studies suggest that histone methylation plays a crucial role in specific human physiological conditions (including cell-cycle regulation, DNA damage and stress response, development and differentiation ^16– 18^) and alterations of this process appear involved in numerous diseases including cancer, cognitive disorders and ageing ^19, 20^. However, research focused around histone methylation components in schistosomes is currently still young with only a few investigations to date suggesting that histone methylation changes are required for life cycle progression ^21– 25^; consequently, schistosome histone methylation machinery components represent emerging new drug targets ^26– 28^.

Previous studies conducted by our research group, as well as others, have explored the druggability of *S. mansoni* lysine specific demethylase 1 (SmLSD1, Smp_150560), a HDM involved in parasite fecundity and motility ^10, 28, 29^. In the current study, we specifically investigated the *S. mansoni* HMT mixed lineage leukemia-1 (SmMLL-1; Smp_138030) homologue as a potential drug target ^28^. As MLL homologues are essential for *Caenorhabditis elegans* in germline stem cell maintenance and fertility ^30^, *Drosophila melanogaster* development ^31^ and *Homo sapiens* haematopoiesis ^32^, developing a drug discovery pipeline centred around SmMLL-1 was rational and additionally well-supported by this schistosome protein’s inclusion in the TDR Drug Target Database ^33^.

Using a combination of functional genomics and structure-based virtual screening (SBVS) methodologies to guide the iterative selection of compounds for entering whole-organism assays, we present evidence that SmMLL-1 is essential to schistosomula survival, adult worm movement, adult worm egg production and miracidia to sporocyst transformation. We additionally demonstrate that the putative SmMLL-1 inhibitors, identified herein, all contain a 1,3,5-triazine core linked to a piperazine ring. As this chemical scaffold has not previously been associated with anti-schistosomal activity, its further medicinal chemistry optimisation could lead to the development of a new class of urgently-needed anthelmintic.

## Methods

### Ethics statement

All procedures performed on mice adhered to the United Kingdom Home Office Animals (Scientific Procedures) Act of 1986 (project license P3B8C46FD) as well as the European Union Animals Directive 2010/63/EU and were approved by Aberystwyth University’s Animal Welfare and Ethical Review Body (AWERB). All animals in this investigation were under the care of a Named Animal Care and Welfare Officer (NACWO), a Named Veterinary Surgeon (NVS), a small animal technician, a personal license holder (PIL) and a project license holder (PPL). While the procedure performed on these mice (infection with *S. mansoni* parasites) is classified within the moderate severity band of our license, efforts to ameliorate harm (outside of that induced by natural parasitic infection) included: provision of environmental enrichment stimulators (ameliorates mental harm), daily welfare and body condition checks (increasing to twice daily at day 45 post-infection) to avoid breaching the severity band of the project license and infection with a minimal number of parasites to reduce the likelihood of developing more than moderately adverse effects.

### Parasite material

The life cycle of the NMRI (Puerto Rican) strain of *S. mansoni* was maintained by routine infections of *Mus musculus* (Tuck Ordinary - TO) female mice and *Biomphalaria glabrata* (NMRI albino and pigmented hybrid) snails. For collection of cercariae, infected snails were exposed to light in an artificially heated room (26°C) for 1 h. The cercarial suspension was subsequently mechanically transformed into schistosomula ^34^ for *in vitro* compound screening or immediately used to percutaneously infect *M. musculus* (180 cercariae/mouse) ^35^ for generation of adult schistosomes. The infected mice (six individuals per cage) were fed with rodents’ diet and water *ad libitum* and housed in a room (temperature 26°C and humidity ~70%) with 12 h light cycle (12 h on, 12 h off). Seven weeks post-infection, experimental animals (18 mice) were euthanised with an intraperitoneal administration of a non-recoverable dose (100 mg/kg) of sodium pentobarbital solution (10 mg/ml, JM Loveridge) containing heparin (100 U/ml solution in 1X PBS, containing 137 mM NaCl, 2.7 mM KCl, 10 mM Na _2_HPO _4_, and 2 mM KH _2_PO _4_). Upon loss of ocular and limb reflex responses to stimuli (4 min), parasite material was obtained by hepatic portal vein perfusion with pre-warmed (37°C) perfusion media (DMEM supplemented with 0.1% 100 U/ml heparin solution in 1X PBS). In brief, the abdominal cavity was opened and the hepatic portal vein was exposed and severed with a 23G needle. This was followed by administration of perfusion media into the left ventricle of the heart to flush worms out of the severed hepatic portal vein. Following perfusion, worms were washed by sedimentation with perfusion media first and then pre-warmed adult worm media (DMEM (Gibco, Paisley, UK) supplemented with 10% v/v FCS (Gibco, Paisley, UK), 1% v/v L-glutamine (Gibco, Paisley, UK) and an antibiotic mixture (150 Units/ml penicillin and 150 µg/ml streptomycin; Gibco, UK)). Following incubation in a humidified environment containing 5% CO _2_ at 37°C for at least 1 h, adult worms were used for either *in vitro* compound screening or RNA interference (RNAi).

Infected livers from perfused mice were homogenised in double saline solution (1.7% w/v NaCl) using a Waring blender and the homogenates were passed through a 0.45 µm filter to retain egg material. Eggs were allowed to hatch in 1X Lepple water ^36^ and the resulting miracidial suspension was enumerated prior to being used for snail infections (12 miracidia/snail) or *in vitro* miracidia to sporocyst screens.

### Homology modelling

The homology model of Smp_138030 was generated within the MOE 2015.10 ^37^ homology tool using a single template approach as previously described ^28^. The program MODELLER could be a valid alternative for comparative protein structure modelling ^38^. The template selected for this protein was the three-dimensional structure of the catalytic domain (SET domain) of *Homo sapiens* MLL3 (PDB ID: 5F6K:C, 58% sequence similarity). An induced fit option was selected to take into account the presence of histone H3, residues 2–7 (the peptide substrate ^39^) and S-adenosyl homocysteine (SAH), the demethylated metabolite of the cofactor SAM. Ten different intermediate models were built and minimised using Amber94 before refining the final model from a Cartesian average of the 10 generated intermediates. The overall quality of the final model was evaluated by RAMPAGE Ramachandran Plot analysis ^40^, ProSA-web ^41^ and Verify3D ^42^.

### RNA interference and quantitative reverse transcription PCR (qRT-PCR)

RNA interference (RNAi), using synthetic short interfering RNAs (siRNAs), was performed as previously described ^10, 28, 43, 44^. Briefly, siRNA duplexes targeting *smp_138030* and non-specific *luciferase* ( *Luc*) were designed and purchased from Sigma (sequences reported in Table 1). Mixed sex adult worms electroporated with a final concentration of 50 ng/μl siRNA duplexes were next cultured at 37°C in adult worm medium (DMEM supplemented with 10% FCS, 2 mM L-glutamine, 10% v/v HEPES (Sigma-Aldrich, UK), 100 Units/ml penicillin and 100 μg/ml streptomycin) in a humidified atmosphere containing 5% CO _2_; a 70% media exchange was performed every 48 h. Adult worms were cultured under these conditions for 48 h prior to assessing *smp_138030* abundance by quantitative reverse transcription PCR (qRT-PCR). An additional set of worms was cultured for 72 h to detect variations in levels of H3K4 methylation in schistosome nuclear extracts/histone preparations. Additionally, a third set of worms was cultured for a total of seven days to monitor adult worm motility daily as well as to analyse fecundity (e.g. egg count). All experiments were replicated three times (15 worm pairs/replicate).

**Table 1.** Small interfering RNA (siRNA) and quantitative reverse transcription PCR (qRT-PCR) oligonucleotide sequences used in this study.

**Target**	**Small interfering RNA (siRNA) sequence**
Smp_138030	CGUUUGGUCCCAUCGGACA[dT][dT] UGUCCGAUGGGACCAAACG[dT][dT]
Luciferase	CUUACGCUGAGUACUUCGA[dT][dT] UCGAAGUACUCAGCGUAAG[dT][dT]
**Target**	**Quantitative Reverse Transcription PCR** **(qRT-PCR)**
Smp_138030	FW: 5´-GTCTACCGGGTGTTCGACG-3´ RV: 5´-TCCAAATCCCGTGCAGC-3´
α-Tubulin Smp_090120 (SmAT1)	FW: 5´-CTTCGAACCAGCAAATCAGA-3´ RV: 5´-GACACCAATCCACAAACTGG -3´

FW: forward; RV: reverse.

For qRT-PCR analyses, 48 h post-siRNA electroporation, worms were homogenised using a TissueLyser LT (Qiagen, UK) in TRIzol Reagent (Invitrogen, UK) before isolation of total RNA using the Direct-zol RNA Kit (R2050, Zymo, UK). cDNA was generated using SensiFAST cDNA synthesis kit (BIO-65053, Bioline) and qRT-PCR was performed with SensiFAST SYBR Hi-ROX mix (BIO-92005, Bioline) and specific qRT-PCR primers (listed in Table 1) for amplifying *smp_138030* and the internal standard *alpha tubulin* ( *smAT1*, *smp_090120*, used as a housekeeping gene) ^45^. The reactions were conducted in a StepOnePlus Real-Time thermal cycler (Applied Biosystems). The comparative threshold Cycle (Ct) mode and Fast protocol was used with the following parameters: 95°C for 20 sec followed by 35 cycles of 95°C for 3 sec followed by 60°C for 30 sec. The resulting data were analysed using the StepOne Software v2.1 (Applied Biosystems) as previously described ^10, 45^.

### Detection of global H3K4 methylation

si *Smp_138030* treated worms were maintained under normal laboratory conditions (5% CO _2_ at 37°C) for three days alongside the si *Luc* controls (five worm pairs per well, 15 worm pairs per condition). Male and female worms were then separately homogenized with a TissueLyser (Qiagen) and total histones extracted using the EpiQuik ^TM ^Total Histone Extraction kit (OP-0006, Epigentek) following the manufacturer’s instructions. The concentration of each sample was quantified by Bradford assay prior to being processed with the EpiQuik ^TM^ Global Histone H3K4 Methylation Assay Kit (P-3017-96, Epigentek) to measure global histone H3K4 methylation levels.

The absorbance (OD) reading at 450 nm of each sample (15 worm pairs per condition, three biological replicates each) was obtained using a POLARstar Omega (BMG Labtech, UK) microtiter plate reader. The percentage of H3K4 methylation was calculated according to the following equation:

$$Methylation\%=\frac{OD\left(sample-blank\right)}{OD\left(\mathrm{untreated control}-blank\right)}\;x\;100\%$$

where the untreated control and the blank were the luciferase controls and the buffer only, (provided in the kit), respectively. The mean of the adjusted control values was set at 100% H3K4 methylation and the standard deviation (SD) was calculated from the normalised values.

### Structure-based virtual screening (SBVS) framework

The structure-based virtual screening (SBVS) framework applied in this study was previously described ^28, 46^ and included four main steps: (1) target and library pre-processing, (2) docking, (3) scoring and (4) post-processing of top-scoring hits. In brief, a library of commercially available compounds (the fragment-based library including 4,352 chemicals) was downloaded from Specs and processed by the Lig Prep tool within Maestro v10.1 ^47^; AutoDock could alternatively be used for this function ^48^. The target model (Smp_138030) was pre-processed using the Protein Preparation Wizard within Maestro (otherwise AutoDock) by assigning bond orders, adding hydrogens and performing a restrained energy minimisation of the added hydrogens using the OPLS_2005 force field. Docking simulations were performed on the substrate binding pocket of the target to evaluate the binding affinity of each Specs compound to a 12 Å docking grid (inner-box 10 Å and outer-box 22 Å) previously prepared using, as a centroid, the substrate peptide.

Initially, the *in silico* molecular docking was performed using the Glide docking software within Maestro (Schrödinger Release 2017 ^47^) using the standard precision function (SP; all 4,532 compounds). AutoDock could be alternatively used as docking program. The results were subsequently refined using the more accurate extra precision (XP) function. The resulting conformations (or poses) of the compounds were ranked according to the Glide XP scoring function with the top 500 distinct compounds identified and retained for a similar docking (both SP and XP) to the corresponding human homologue *H. sapiens* MLL3 (PDB ID: 5F6K), previously prepared as described above for the schistosome protein. As a result, each compound was associated with a pair of docking scores (the SP and XP scoring function) for both the schistosome and the corresponding human homologue. The compounds with a more favourable docking score (i.e. the lower energy value represented by more negative values for both XP and SP scores) for the parasite protein compared to the human template were selected. A subset of compounds (defined here as first set of compounds) was carefully chosen to encompass maximal chemical diversity and purchased for biological screens. Refinement of the first selection criterion led to the identification of chemicals (defined here as second set of compounds) having a more favourable docking score for the parasite protein compared to the human template for at least one of the scoring function (either SP or XP score).

In a final stage of the study, we used the central scaffold of compound 7 (6-(piperazin-1-yl)-1,3,5-triazine-2,4-diamine, simplified molecular-input line-entry) as query structure to search for any remaining structural analogues of compound 7 present in the Specs fragment-based library. This structure-based search resulted in 17 compounds, of which nine chemicals were already included in the second set of compounds. The remaining eight small molecules were purchased and screened to fully explore the chemical space around the central scaffold of compound 7. These chemicals were not previously identified because they did not have a more favourable docking score (neither SP nor XP score) for the parasite protein compared to the human template. Overall, this approach exhaustedly investigated the whole Specs database for any closely structural related analogues of compound 7.

### Schistosomula screens

Anthelmintic activity on the schistosomula stage of *S. mansoni* was evaluated using Roboworm, an integrated high-throughput, high content image analysis platform originally developed by Paveley *et al.* ^49^ and subsequently used by our research group as previously described ^10, 28, 50, 51^. Alternatively, the computer application Worminator can be used to assess motion of microscopic parasites such as the schistosomula stage of *S. mansoni* ^52^. Compounds (as single concentration or two-fold titrations) were prepared for screening in a 384-well plate (PerkinElmer, MA, USA), where solutions (1.6 mM, 0.5 μl) were wet stamped using the Biomek NX ^P^ liquid handling platform into Basch medium (20 μl) ^53^. Negative (0.625% DMSO) and positive (Auranofin - AUR - at 10 µM final concentration in 0.625% DMSO) control wells were similarly prepared. Mechanically transformed schistosomula (about 120 parasites in 60 μl of Basch medium) were distributed into each treated well using a WellMate (Thermo Scientific, Loughborough, UK). Schistosomula/compound co-cultures were then incubated at 37°C for 72 h in a humidified atmosphere containing 5% CO _2_. At 72 h, parasite/compound co-cultures were resuspended and tissue culture plates were imaged under the same conditions (37°C for 72 h in a humidified atmosphere containing 5% CO _2_) using an ImageXpressXL high content imager (Molecular Devices, UK) with subsequent images processed for phenotype and motility as previously reported ^49^. Plate analysis returned a phenotype and motility score for each well and any scores below -0.15 and -0.35 (defined threshold anti-schistosomula values for phenotype and motility scores, respectively) defined a condition displaying anti-schistosomula activity. Compound screens were assessed based on the Z´ values (derived from the means and standard deviations of positive and negative controls ^54^) and those equal to or above 0.3 for both phenotype and motility were considered successful.

Phenotype and motility scores of each titration were used to generate dose response curves in GraphPad Prism 7.02 based on the corrected average score of the three replicates for each data point. These data were then used to calculate EC _50_ (the concentration of compound that caused motility or phenotype defects in 50% of the treated parasite when compared to untreated controls) values.

### Adult worm screens

For compound screening, adult worms (three worm pairs/well for compound 7 and the hit compounds of set two; one worm pair/well for the hit compounds of set three) were transferred into wells of a 48-well tissue culture plate containing 1 ml of adult worm media. Adult worms were treated with compounds previously identified as hits at 10 µM on 72 h cultured schistosomula. Worms were dosed with an appropriate volume of 10 mM stock solution of each compound (in DMSO) to have a concentration range of 50 – 6.25 µM (up to 0.5% DMSO, primary screen of compound 7). Secondary screens were performed on the structural analogues of compound 7 (only the schistosomula hits at 10 µM) at a single concentration (12.5 µM) and compared to the same concentration of the parental compound (included in the screen as reference). DMSO (0.5%) and praziquantel (10 µM in 0.5% DMSO) were also included as negative and positive control treatments (both primary and secondary screens). Treated adult worms were incubated for 72 h in a humidified environment at 5% CO _2 _at 37°C. Parasite motility after drug treatment was assessed by a digital image processing-based system (WormassayGP2, see *Software availability* ^55^) modified after Wormassay ^52, 56, 57^. Egg production was assessed according to the methodology described by Edwards *et al.* ^58^.

### Miracidia screens

Following hatching of *S. mansoni* eggs, miracidia were incubated on ice for 15 min and then centrifuged at 700 x *g* for 5 min at 4°C. The miracidia pellet was then re-suspended in 5 ml of Chernin's balanced salt solution (CBSS), subjected to pelleting and two subsequent washes (all at 700 x *g* for 5 min at 4°C). Afterwards, the supernatant was carefully removed with a serological pipette and the miracidia-enriched pellet was resuspended with CBSS supplemented with 1 mg/ml each of glucose and trehalose and 500 µl of Penicillin-Streptomycin (containing 10,000 units penicillin and 10 mg streptomycin/ml, P4333, Sigma-Aldrich) ^23^. In preparation for the screens, CBSS (250 µl) was aliquoted to each well of a 24-well plate followed by addition of each compound in DMSO (1 mM concentration in 1% DMSO) or DMSO alone (1%, negative control). Next, approximately 20-25 miracidia (in 250 µl of CBSS) were aliquoted to each well. A preliminary dose-response titration of compound 7 (50, 25, 10, 5, 2 and 0.5 µM in 1% DMSO) was performed and secondary single-concentration (10 µM in 1% DMSO) screens of the most active (hits on schistosomula at 10 µM) structural analogues followed. Both preliminary and secondary screens were performed in duplicate on two separate occasions (two independent biological replicates). Miracidia were incubated for 48 h at 26°C before being evaluated using an Olympus inverted light microscope for morphological and behavioural changes differing from the control wells. Dead, partially transformed and fully transformed miracidia were enumerated in the DMSO control and the assay plates as previously described ^5, 23, 59^. In brief, parasites were visually scored “dead” if no macroscopic movement and flame-cell activity was detected and tegument/epidermal surface showed signs of degeneration. “Transforming” parasites were no longer swimming, had a “rounded” morphology and were in the process of shedding epidermal plates. Absence of ciliated plates attached to the surface of the parasite and formation of sporocyst tegument defined the so-called “fully transformed” parasites.

### Cytotoxicity assay on human surrogate cell line (HepG2 cells)

Compound cytotoxicity on Human Caucasian Hepatocyte Carcinoma (HepG2) cells (85011430, Sigma Aldrich) was elucidated by the MTT (3-(4,5-dimethylthiazol-2-yl)-2,5-diphenyltetrazolium bromide) tetrazolium reduction assay as previously described ^10, 28, 50^. Briefly, HepG2 cells were seeded at a density of 20,000 per well in 96-well black, clear bottom falcon plate in modified BME medium (50 μl; containing 1% antibiotic/mycotic (Sigma-Aldrich, Gillingham, UK), 1% 200 mM L-glutammine (Gibco, Paisley, UK), 1% MEM non-essential amino acid solution (Gibco, Paisley, UK), and 10% fetal bovine serum (Gibco, Paisley, UK)). HepG2 plates seeded for cytotoxicity screens were treated with compounds 24 h post-seeding. Each compound was tested at 200, 100, 75, 50, 20, 10 and 1 µM; each concentration was assayed in triplicate. Blank wells (no cells), as well as positive (1% v/v Triton X-100, X100, Sigma-Aldrich) and negative (1.25% v/v DMSO or media only) controls, were included in each plate. After addition of compounds, each plate was then incubated for a further 20 h before application of MTT reagent for assessment of compound cytotoxicity (4 h incubation with MTT for a total 24 h cell-drug incubation) ^60^. The absorbance reading at 570 nm was measured with a POLARstar Omega (BMG Labtech, UK) microtiter plate reader. Dose response curves were generated in GraphPad Prism 7.02 based on the corrected average absorbance of the three replicates for each concentration point. These data were then used to calculate CC _50_ (the concentration of compound that reduced cell viability by 50% when compared to untreated controls) values.

### Scanning electron microscopy to assess compound damage on adult worms

Following 72 h of drug treatment, the cultured *S. mansoni* adult worms were collected, separated by sex and relaxed with tricaine anaesthetic solution (0.25% w/v of ethyl 3-aminobenzoate methane sulfonate - Tricaine, E10521, Sigma Aldrich - in DMEM). The samples were incubated by gently rocking for 15 min or until parasites relaxed and separated. The parasites were then killed by incubation in a solution of 0.6 mM magnesium chloride (MgCl _2_) for 1 min. Afterwards, adult schistosome worms were briefly washed with pre-warmed 1X PBS and then fixed using appropriate solutions for analysis by scanning electron microscopy (SEM) as previously described in literature ^58, 61, 62^.

Post fixation, adult schistosomes were stored at 4°C until washing with 0.1 M sodium cacodylate was performed (2 x 30 min each). The worms were then stained with 1% v/v osmium tetroxide water solution (stored at -20°C and pre-warmed at room temperature prior to use) for 2 h and then washed with 0.1 M sodium cacodylate for 30 min. Following staining, worms were dehydrated with an aqueous alcohol series (30, 50, 70, 95 and 100% ethanol in ultra-pure water) by agitating gently on a rocker for 30 min per solution. At the final step, the samples were left in 100% ethanol overnight before being dried with hexamethyldisilazane critical drying point agent (AGR1228, Agar Scientific, Stansted, UK) for at least 3 h.

Following critical point drying, the organic solvent was removed with a Pasteur pipette and the samples were left to dry overnight. Samples were then placed onto self-adhesive conductive carbon tabs on aluminium specimen stubs (both Agar Scientific, Stansted, UK) and then were coated with gold using a Polaron E5000 SEM Coating Unit. Coated worms were then stored in a desiccation jar until imaging on a Hitachi S-4700 FESEM microscope using the Ultra High-Resolution mode and an accelerating voltage of 5.0 kV at a working distance of 5.0 mm.

### Statistical analysis

All statistical analyses were performed using GraphPad Prism 7.02. In detail, comparisons between two or multiple groups were performed using Mann-Whitney U-test (Student’s *t* test, two tailed, unequal variance) and Kruskal-Wallis test followed by Dunn’s test, respectively.

## Results

### Homology model of Smp_138030

**Figure 1.**
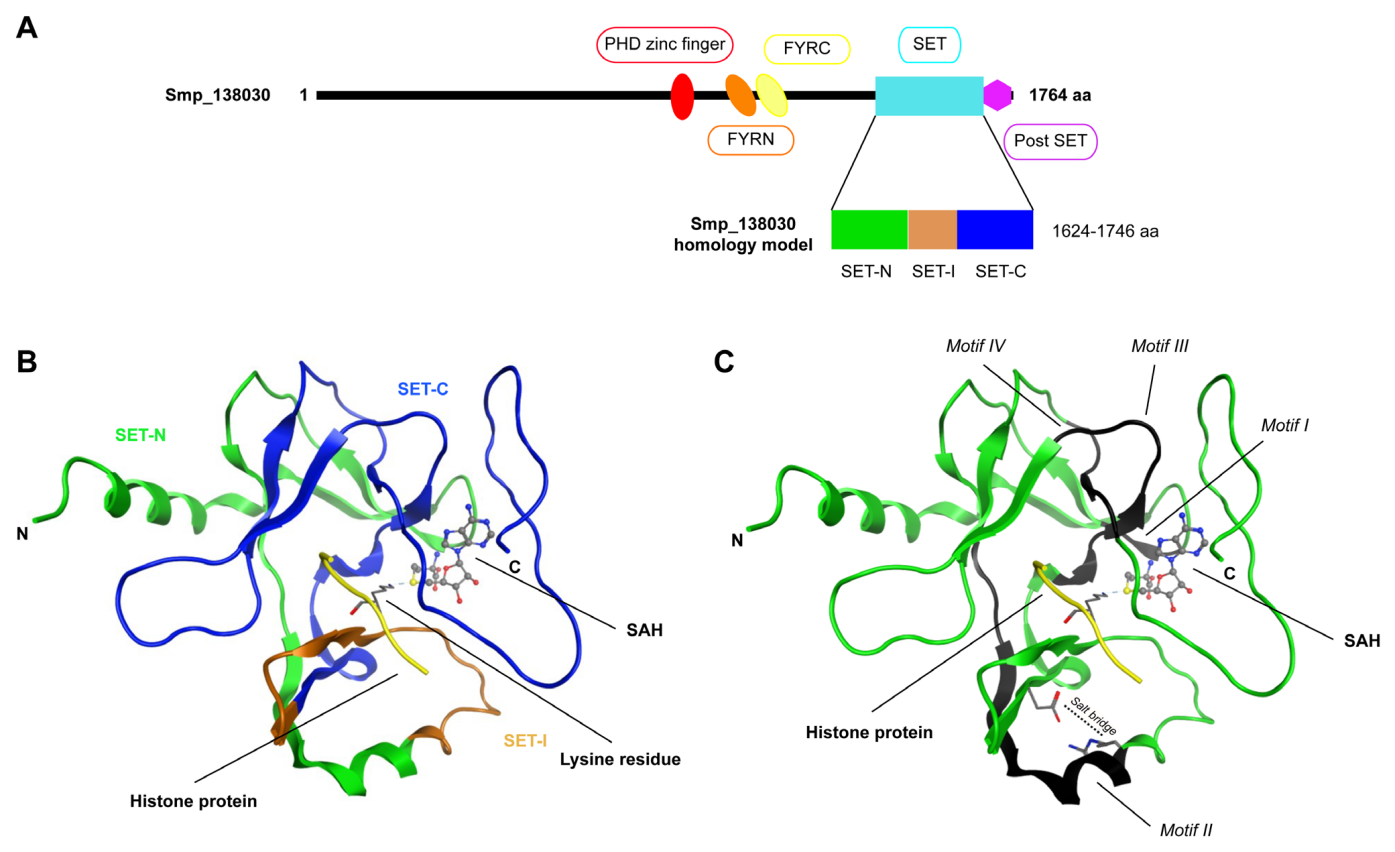
Smp_138030/SmMLL-1 is a SET domain containing protein lysine methyltransferase (PKMT). **Panel A**- Schematic representation of the domain architecture of Smp_138030 is reported with indication of the different domains. PHD zinc finger - Plant homeodomain zinc finger; FYRN - FY-rich domain N-terminal; FYRC - FY-rich domain C-terminal; Post-SET - post Su(var)3-9, Enhancer-of-zeste and Trithorax domain. A focus of the SET domain contained in the homology model is provided: SET-N and SET-C domains (in green and blue, respectively) linked by a third domain (SET-I, in light brown). **Panel B** - Ribbon drawing of the structure of Smp_138030’s SET domain constructed using the homology modelling approach shows the different structural elements (SET-N, SET-I and SET-C) highlighted in different colours (same colour code used for Panel A). **Panel C** - Same orientation as in B, but highlighting the structural motifs I to IV in black. The N- and C-termini of the protein are labelled as ‘N’ and ‘C’, respectively. S-adenosyl homocysteine (SAH) is shown as spheres and sticks (grey for carbons, red for oxygen, blue for nitrogen); the peptide from histone H3 is represented as yellow ribbon (the methylated lysine is shown as grey stick). In Panel C, the two residues (the conserved arginine of Motif II and a conserved glutamic acid in the SET-C region) responsible of the salt bridge are shown in stick mode. All hydrogens are removed from all residues and chemical structures for clarity. Graphical representation of Panel A is created using IBS 1.0; images of Panel B and C are created using MOE 2015.10. The program MODELLER could be a valid alternative for comparative protein structure modelling.

Schistosome genome analyses predicted a full set of SmHMTs and SmHDMs cooperatively regulating histone methylation in *S. mansoni* ^21, 28^. Among the 20 identified protein lysine methyl transferases (PKMTs), Smp_138030 was characterised as the closest homologue of the human mixed lineage leukemia (MLL) HMT; this schistosome protein will be referred to as SmMLL-1 from this point forward ( Figure 1). SmMLL-1 contains a SET domain, a post SET domain and FY-rich N/C terminal (FYRN and FYRC) sequence motifs ( Figure 1A), which are particularly common in histone H3K4 methyltransferases like MLLs ^63^. SmMLL-1’s homology model was constructed using the catalytic domain (SET domain) of the human template MLL3 ( Figure 1B). The global quality of each model was validated by Ramachandran plot analysis, ProSA-web and Verify 3D (Supplementary Figure 1, *Extended data* ^64^); all approaches surpassed agreeable standards defined in the literature ^41, 65^. Overall, these tools suggested that Smp_000700/SmMLL-1’s homology model was of reasonable quality compared to the human MLL3 template and, thus, suitable for further experiments.

SmMLL-1’s SET domain (defined by SET-N, SET-I and SET-C, Figure 1A) contains a series of β stands, which fold into three discrete sheets around a unique knot-like structure ( Figure 1B) ^66^. In this structure, the SET domain C-terminus threads through a loop region, which is formed by a hydrogen bond between two segments of the protein chain ^67^. The homology model of this PKMT shows the position of the four conserved motifs within the SET domain ^67^. Motifs I and II are found within the SET-N domain, whereas motifs III and IV are found within the SET-C domain. Motif I (or GxG motif where x represents any amino acid, usually bulky hydrophobic residues) defines the cofactor binding pocket and most of the conserved residues of this motif are mainly associated with cofactor binding. Motif II is involved in the catalytic activity of these SET proteins and its arginine residue forms a salt bridge with a conserved glutamic acid in the SET-C region ( Figure 1C) contributing to the structural stability of the protein. Motif III, containing the RFINHSCxPN sequence (where x represents any amino acid), is responsible for substrate recognition as well as the formation of the pseudoknot structure ^66^ with Motif IV (containing the GEELxxDY consensus sequence, where x represents any amino acid) located close to the C-terminus ( Figure 1C).

### Functional genomics investigation of Smp_130830/SmMLL-1

**Figure 2.**
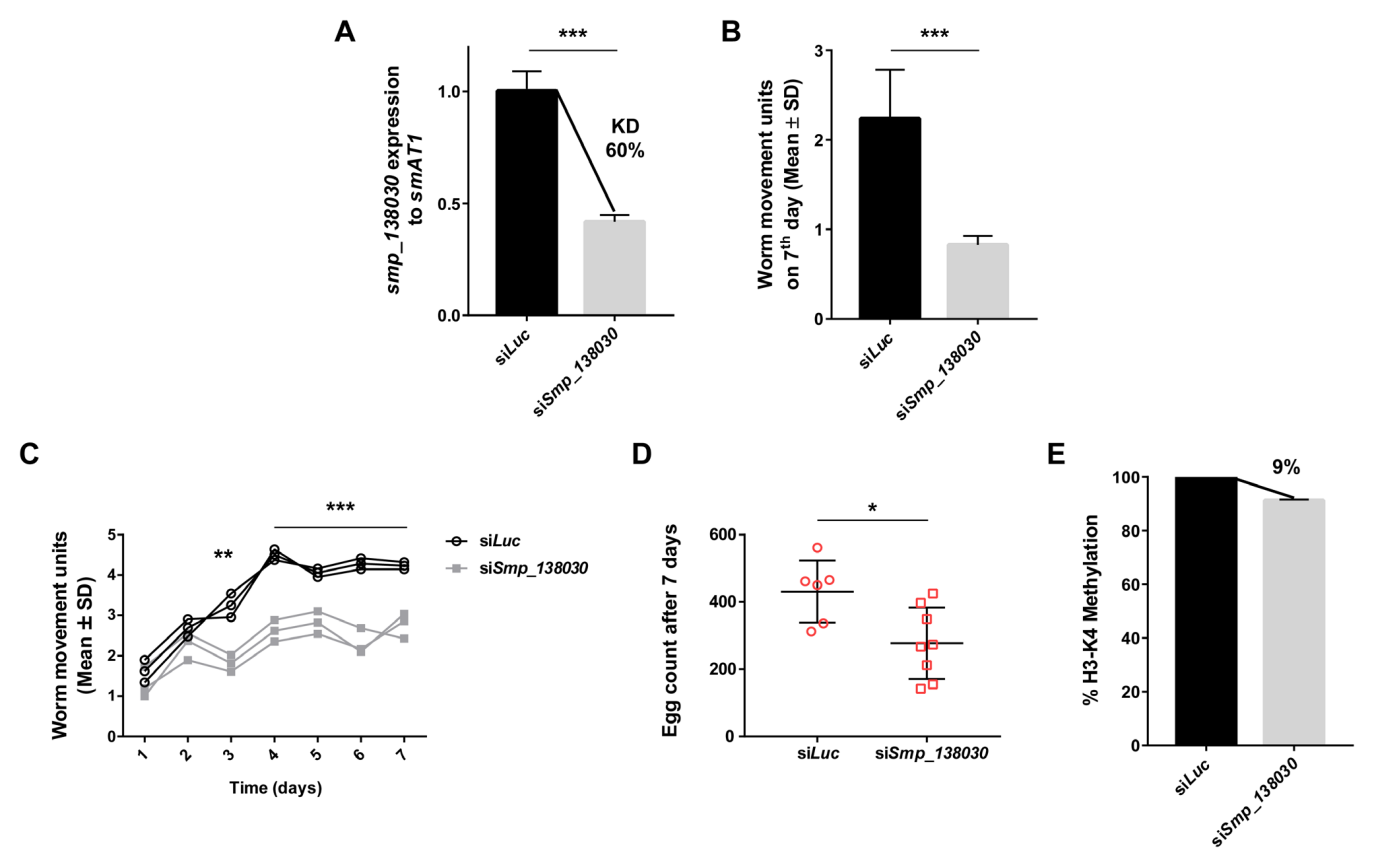
RNAi-mediated knockdown of *smp_138030/smmll-1* affects worm movement, *in vitro* production of schistosome eggs and H3K4 methylation. **Panel A** - Seven-week old adult male and female schistosomes were electroporated with 5 μg siRNA duplexes targeting *luciferase* (si *Luc*) or *smp_138030* (si *Smp_138030*). Following 48 h, total RNA was harvested and subjected to qRT-PCR. Percent knockdown (KD) and statistical significance (Student’s *t* test, two tailed, unequal variance - Mann-Whitney U-test) is indicated. All siRNA and qRT-PCR DNA sequences are included in Table 1. **Panel B** - Five worm pairs for each treatment (si *Luc* and si *Smp_138030*, repeated three times; n = 15 per sex per treatment) were cultivated for seven days post treatment in an atmosphere of 5% CO _2_ with a 70% media exchange performed every 24 h. Quantification of worm movement was performed using WormassayGP2 (see *Software availability* ^55^) on the 7th day. **Panel C** - Daily movement of siRNA treated worms quantified by WormassayGP2. **Panel D** - Egg production at 168 h after introduction of siRNAs. **Panel E** - Following RNAi-induced *smp_138030* knockdown, total histone extracts from RNAi targeted worms (si *Smp_138030*) and control worms (si *Luc*) were analysed for H3K4 methylation. The bar chart represents the average H3K4 methylation detected in male and female pairs as there was no statistical significance between sexes. Statistical significance is indicated (Student’s *t* test, two tailed, unequal variance - Mann-Whitney U-test). *, ** and *** represent *p* < 0.05, *p* < 0.001 and *p* < 0.001, respectively.

RNAi-mediated post-transcriptional gene silencing was employed to explore the biological role of this SET-domain containing protein in adult worm motility and fecundity ( Figure 2). qRT-PCR assessment of *smp_138030* transcript abundance in si *Smp_138030* treated worms revealed a decrease of 60% when compared to si *Luc* treated worms at 48 h post-electroporation ( Figure 2A). Despite some signs of distress immediately after electroporation, all worms recovered and appeared robust up until seven days post-treatment (the last time point examined in this study). Examination using light microscopy showed no specific phenotypic differences between the luciferase control and the target RNAi worms; no death was observed, all parasites retained gut peristalsis (as indicated by haemozoin movement) but they partially lost adherence to the tissue culture wells. However, movement was significantly reduced in si *Smp_138030* treated worms at this time-point when compared to si *Luc* controls ( Figure 2B). Daily measurements of worm movement were next assessed starting at 24 h post electroporation and continuing until day seven ( Figure 2C). By doing so, we observed a significant difference in worm movement between the control and the treated worms starting at day three and continuing up until day seven. In addition to this motility defect, *smp_138030* knockdown also led to anti-fecundity effects. In fact, at day seven post siRNA treatment, egg production was significantly inhibited (~40% reduced) in si *Smp_138030* treated worms ( Figure 2D). To assess whether a 60% *smp_130830* knockdown resulted in a histone methylation defect in adult schistosomes, an ELISA-based assay quantifying methylation on histone H3, lysine 4 (H3K4) was performed on adult worm (both male and female) histone protein extracts. These assays revealed that *smp_138030* knockdown resulted in a 9% reduction (not statistically significant) of H3K4 methylation ( Figure 2E).

### The identification of putative SmMLL-1 inhibitors and their activity on schistosomes

**Figure 3.**
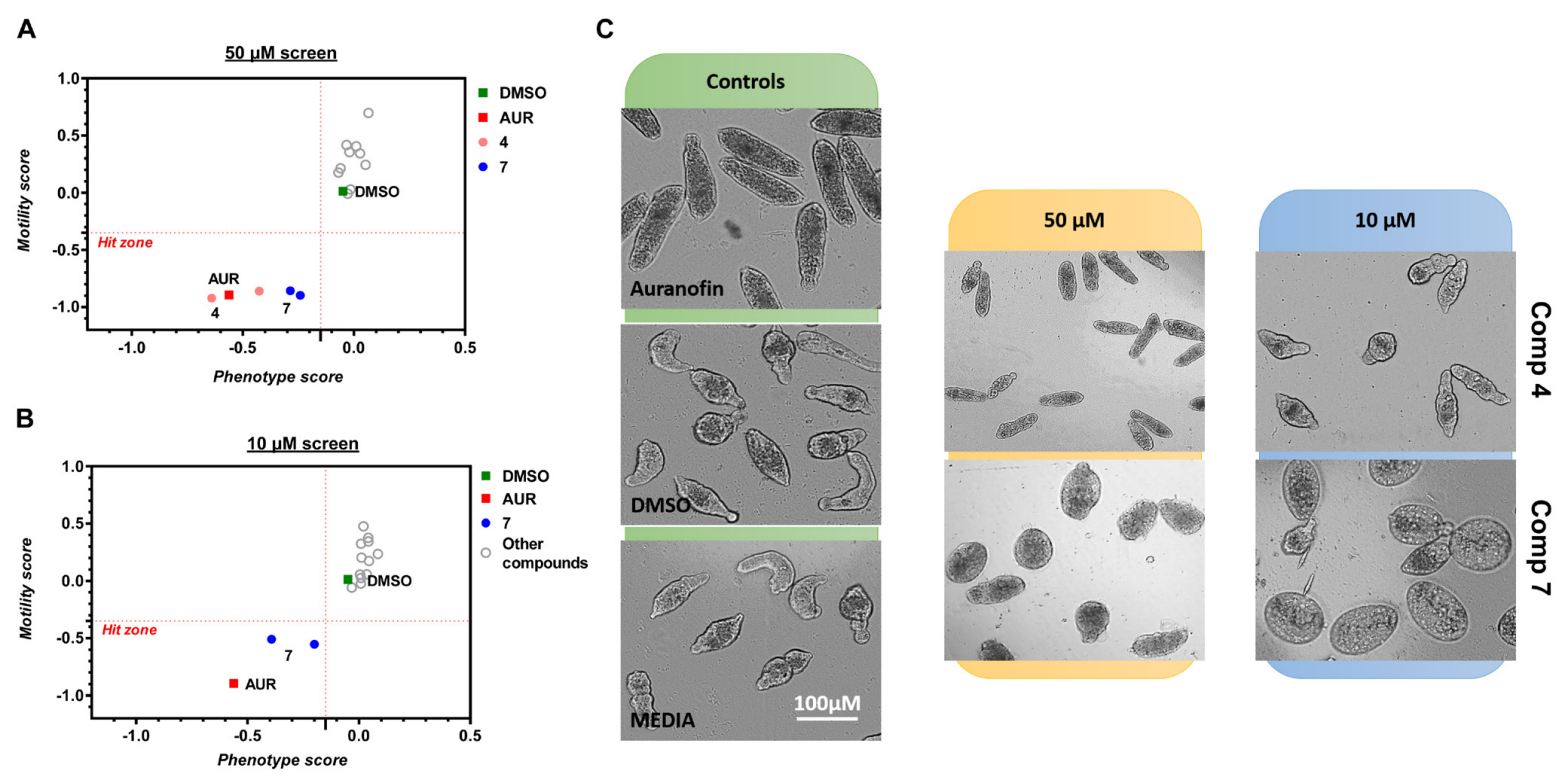
Putative SmMLL-1 substrate-binding pocket inhibitors (set one) affect schistosomula phenotype and motility. Mechanically-transformed schistosomula (n = 120) were incubated with the selected compounds (50 µM - **Panel A** - and 10 µM - **Panel B** - in 0.625% DMSO; each of them in duplicate) for 72 h at 37°C in a humidified atmosphere containing 5% CO _2_. One of three replicate screens is shown here (barcode 0255, Supplementary Table 2, *Extended data* ^64^). Compounds with activity on both schistosomula phenotype and motility are shown within the ‘Hit Zone’ (delineated by the dotted red lines in the graph). **Panel C** - Visual representations of schistosomula phenotype induced by compounds 4 and 7. These two compounds were tested on schistosomula at 50 and 10 µM. Each image represents a cropped sample of the schistosomula obtained from hit wells of screen 0255. The brightness of the image has been modified slightly (+ 20%) from the original image to make the differences in phenotype more apparent. Images of control parasites - Auranofin, DMSO and media treated schistosomula - are included for reference.

After establishing that SmMLL-1 was a druggable target by RNAi, a collection of seven compounds (Supplementary Table 1, *Extended data* ^64^) with a more favourable docking score (both SP and XP score) for SmMLL-1 compared to the human template (MLL3) were screened for anti-schistosomula activity at 50 and 10 µM ( Figure 3). The calculated Z´ values for both phenotype and motility of the performed screens (summarised in Supplementary Table 2, *Extended data* ^64^) were within accepted ranges as previously described ^54^. Among these compounds, two (compounds 4 and 7, Table 2) showed anti-schistosomal activity ( Figure 3A) at 50 µM with only compound 7 remaining active at 10 µM ( Figure 3B). At 50 µM, compound 4 treated parasites presented an elongated phenotype similar to the Auranofin-treated schistosomula ( Figure 3C). In contrast, compound 7 treated schistosomula (at both 50 and 10 µM) appeared swollen with some individuals additionally displaying posterior end compression (as shown in Figure 3C). A secondary anti-schistosomula titration of compound 7, across four independent screens (Z´ values listed in Supplementary Table 2, *Extended data* ^64^), was subsequently performed to quantify its potency; here, EC _50 _values of 5.65 µM for phenotype and 5.03 µM for motility were obtained (Supplementary Figure 2, *Extended data* ^64^).

**Table 2.** Docking results of human and schistosome proteins with the two selected compounds.

Compound	Structure	Binding affinity with schistosome target (kcal/mol)		Binding affinity with human target (kcal/mol)	
		SP	XP	SP	XP
**4**	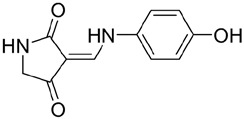	-6.279	-7.058	-5.981	-5.909
**7**	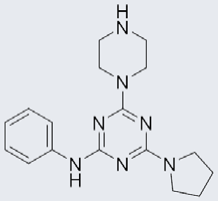	-6.297	-7.86	-5.018	-5.176

Compound 4 and 7 were docked to the schistosome Smp_138030 and the closest human homologue (MLL3, PDB ID: 5F6K). SP - standard precision; XP - extra precision.

**Figure 4.**
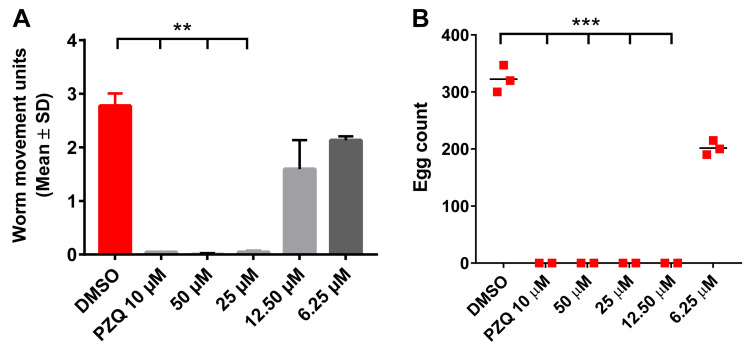
The most potent anti-schistosomula compound (7) affects adult worm motility and egg production. A dose response titration (50 - 6.25 µM) of compound 7 was performed to assess its potency on *S. mansoni* adult worms (three worm pairs/well for each condition). **Panel A** - Schistosome motility was quantified using WormassayGP2 at 72 h. **Panel B** - Eggs were collected and enumerated by brightfield microscopy at 72 h. Each titration was performed in three independent screens. A Kruskal-Wallis ANOVA followed by Dunn’s multiple comparisons test was performed to compare each population mean to DMSO mean value. ** and *** represent *p* < 0.0055 and *p* < 0.0003, respectively.

The anti-schistosomal activities of compound 7 was next explored on adult worms ( Figure 4). At 50 and 25 µM, the compound had a lethal effect, however, worm recovery began at 12.50 µM and remained at 6.25 µM ( Figure 4A). In terms of egg production, compound 7 was particularly effective in inhibiting worm fecundity up to and including 12.50 µM ( Figure 4B).

Scanning electron microscopy was used to evaluate the morphological changes induced by *in vitro* treatment of the parasite with a sub-lethal concentration (12.50 µM) of compound 7 (Supplementary Figure 3A, B and C, *Extended data* ^64^) compared to the control (Supplementary Figure 3D, E and F, *Extended data* ^64^). The compound induced partial loss of tubercles and spines as well as tegument erosion (asterisks in Panel B) and ulcerations (red arrows in Panel C).

### Anti-schistosomal activity of compound 7 structural analogues

Based on these preliminary data which led to the identification of compound 7, the results of the comparative docking study of the Specs library were subsequently explored with a less stringent criterion in this second stage of the study. This led to the identification of a second set of compound 7 analogues (see Supplementary Table 3, *Extended data* ^64^) with a better predicted binding affinity to the substrate binding site of SmMLL-1 compared to its closest human homologue MLL3 (PDB ID: 5F6K), based either on SP or XP docking score (i.e. at least one scoring function, conversely to the first selection of compounds where both conditions had to be satisfied).

**Figure 5.**
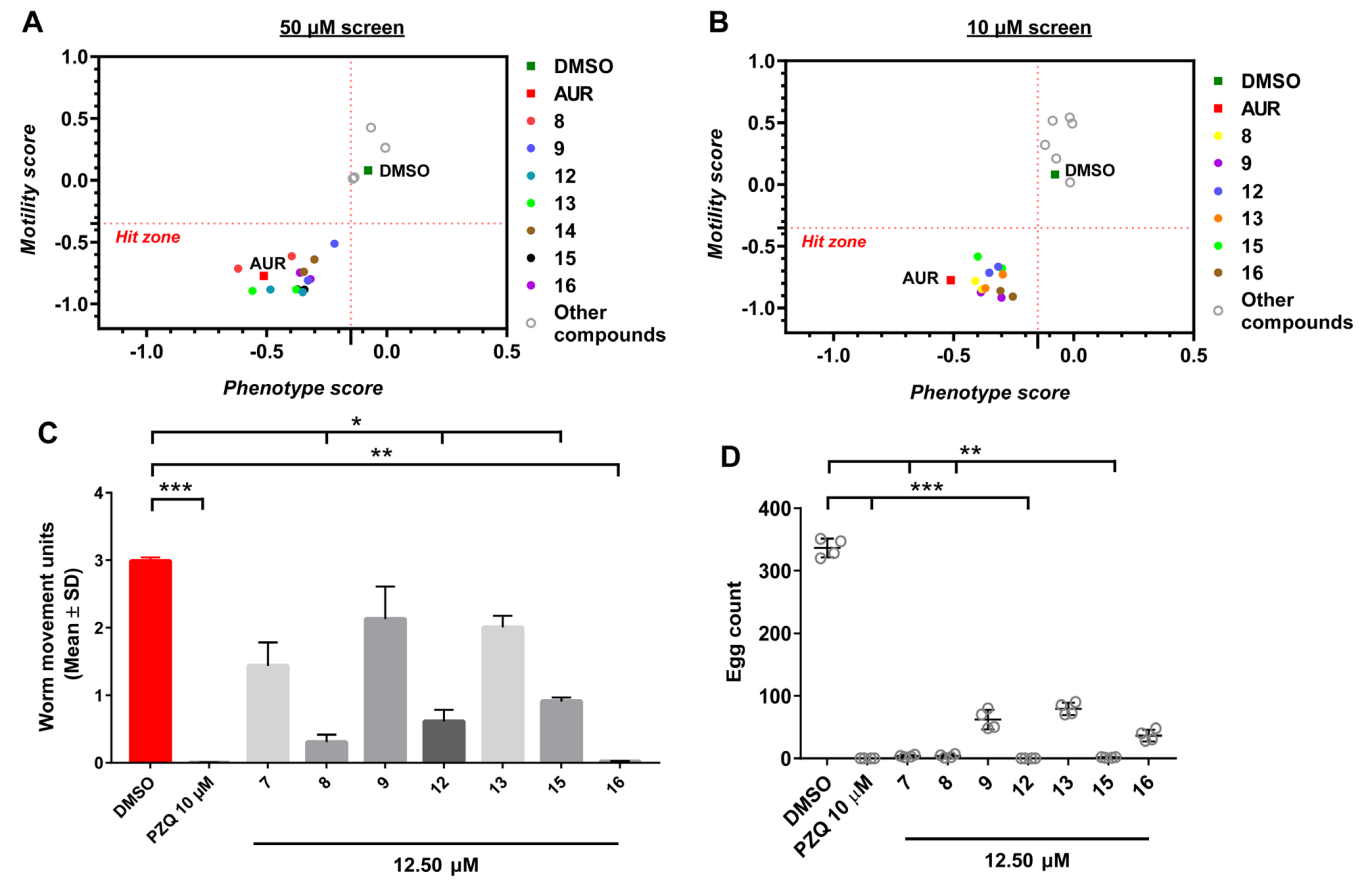
Compound 7 structural analogues (set two) demonstrate more potent anti-schistosomal properties. Mechanically-transformed schistosomula (n = 120) were incubated with the selected compounds (50 µM - **Panel A** - and 10 µM - **Panel B** - in 0.625% DMSO; each of them in duplicate) for 72 h at 37°C in a humidified atmosphere containing 5% CO _2_. One of three replicate screens is shown here (barcode 0329, Z´ scores are reported in Supplementary Table 4, *Extended data* ^64^). The screen and the interpretation of the data is described in Figure 3. Compounds with activity on both schistosomula phenotype and motility are shown within the ‘Hit Zone’ (delineated by the dotted red lines in the graph). **Panel C** - Six compounds (8, 9, 12, 13, 15 and 16) were incubated with adult worms (three worm pairs/well for each condition) at 12.5 µM (sublethal concentration for 7, here reported as reference) to assess their effect on motility at 72 h. Each screen was performed in duplicate in two independent screens. The effect on schistosome motility was quantified using WormassayGP2 (see *Software availability* ^55^). **Panel D** - After 72 h of drug treatment, eggs were enumerated. The egg count for each concentration tested is reported in the scatter chart. For each concentration tested, the mean egg count and the standard error across the two biological replicates with two technical replicates each is represented on the graph. Statistical analysis was performed similarly to Figure 4. *, **, *** represent *p* < 0.0208, *p* < 0.0055, *p* < 0.0003, respectively.

This second compound family encompassed nine structural analogues of compound 7 and these were initially tested on schistosomula at 50 and 10 µM ( Figure 5, Z´ values summarised in Supplementary Table 4, *Extended data* ^64^). Six out of nine tested compounds were hits on the larva stage of the parasite at both 50 and 10 µM ( Figure 5A and B), except for compound 14, which was only active at 50 µM. All compound 7 analogue hits at 10 µM were tested at lower concentrations to assess their anthelmintic potencies; EC _50_ values were derived from these dose response titrations ( Table 3).

**Table 3.** Biological properties of compound 7 and its structural analogues.

	Schistosomula EC _50_ (µM)			Adult EC _50_ (µM)	CC _50_ (µM)
Compound	Phenotype	Motility	Average		HepG2 cells
**7**	5.65	5.03	5.34	11.02	13.65
**8**	3.27	4.98	4.12	N.D.	14.88
**9**	3.39	3.6	3.50	N.D.	28.34
**10**	> 50			N.D.	N.D.
**11**	> 50			N.D.	N.D.
**12**	4.57	4.41	4.49	N.D.	10.98
**13**	4.70	4.82	4.76	N.D.	17.86
**14**	> 10			N.D.	N.D.
**15**	1.09	1.79	1.44	N.D.	23.86
**16**	2.63	3.37	3.00	N.D.	54.18
**17**	> 10			N.D.	N.D.
**18**	> 50			N.D.	N.D.
**19**	> 50			N.D.	N.D.
**20**	3.47	4.93	4.20	N.D.	>100
**21**	> 50			N.D.	N.D.
**22**	2.09	1.64	1.87	N.D.	93.55
**23**	3.06	2.79	2.92	N.D.	6.73
**24**	1.57	1.81	1.69	N.D.	3.31

Schistosomula EC _50_ (phenotype, motility and average of both metrics) values are calculated based on three dose response titrations (10 - 0.625 µM). Where no effect is seen on schistosomula at the highest concentration tested (50 µM), the EC _50 _is said to be more than 50 µM. The EC _50 _is said to be more than 10 µM if the compound is defined as a hit at 50 µM but not at 10 µM. Adult worm EC _50_ value is calculated based on two dose response titrations (100 – 3.13 µM). The CC _50_ values are calculated based on three dose response titrations (200 - 1 µM) using the HepG2 cell line. All data are analysed using GraphPad Prism. N.D.: not determined.

Among this second family of compound 7 analogues, only the small molecules demonstrating anti-schistosomula activity at 10 µM were subsequently screened against adult worms ( Figure 5C). Specifically, they were only tested at 12.50 µM (the sublethal concentration for compound 7) in order to identify those with improved anthelmintic activity over compound 7. This comparative approach identified four compounds (compound 8, 12, 15 and 16) as more potent than the original hit compound. Moreover, all tested compounds demonstrated a negative effect on schistosome fecundity with compounds 8, 12 and 15 reducing egg production similarly to compound 7 ( Figure 5D).

### Anti-schistosomal activity of remaining compound 7 structural analogues

**Figure 6.**
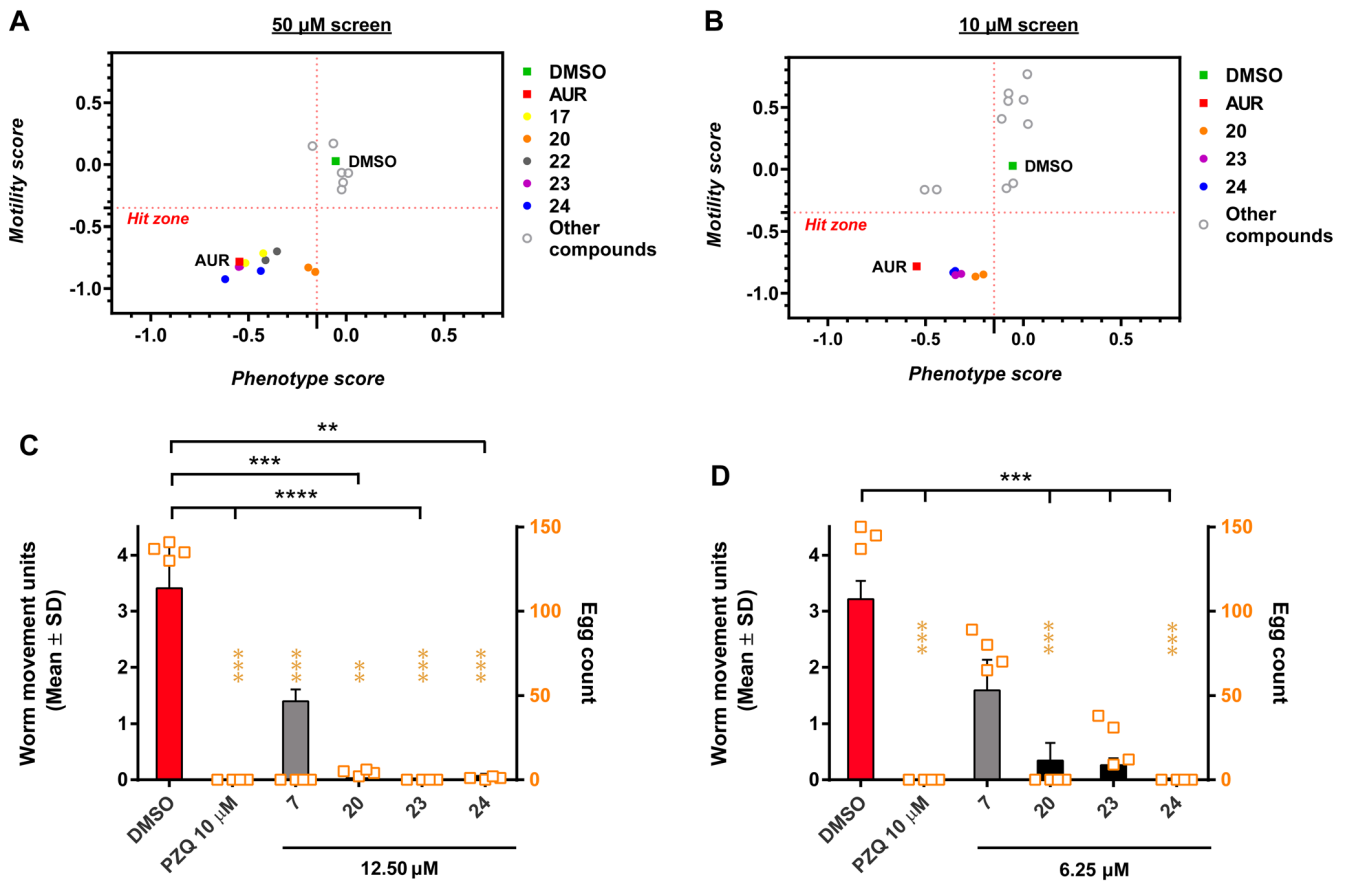
Anti-schistosomal activity of the remaining compound 7 analogues found within the Specs fragment-based library. Mechanically-transformed schistosomula (n = 120) were incubated with the selected compounds (50 µM - **Panel A** - and 10 µM - **Panel B** - in 0.625% DMSO; each of them in duplicate) for 72 h at 37°C in a humidified atmosphere containing 5% CO _2_. One of the three independent performed screens is shown here as a representative of the preliminary larva screen (barcode 0358, Z´ scores are reported in Supplementary Table 6, *Extended data* ^64^). The screen and the interpretation of the data is described in Figure 4. Compounds with activity on both schistosomula phenotype and motility are shown within the ‘Hit Zone’ (delineated by the dotted red lines in the graph). Three compounds (20, 23 and 24) were tested on adult worms at 12.5 µM (sublethal concentration for compound 7, here reported as reference, **Panel C**) and 6.25 µM ( **Panel D**) to assess their potency on *S. mansoni* adult worms (one worm pair/well for each condition). Each screen was performed in duplicate in two independent screens. The effect on schistosome motility was quantified as described above using WormassayGP2. After 72 h of drug treatment with 3 structural analogues at 12.5 µM (scatter plot, **Panel C**) and 6.25 µM (scatter plot, **Panel D**), eggs were enumerated. The hit compound 7 is included in the screen as reference. The egg count for each concentration tested is reported as a scatter chart. For each concentration tested, the mean egg count and the standard error across the two biological replicates with two technical replicates are represented on the graph. Statistical analysis (in black for the motility, in orange for the egg count) was performed similarly to Figure 4. **, ***, **** represent *p* < 0.0055, *p* < 0.0003, *p* < 0.0001, respectively.

In a final stage of the study, any other remaining compounds commercially available through Specs containing the central compound 7 scaffold (using 6-(piperazin-1-yl)-1,3,5-triazine-2,4-diamine as query structure in Specs database) were next selected (without any docking score-based selection, Supplementary Table 5 *, Extended data* ^64^) and tested on *in vitro* cultured schistosomes ( Figure 6). This third set of compounds was initially tested on schistosomula and, while five chemicals (17,20, 22, 23 and 24) were active at the highest concentration (50 µM) ( Figure 6A), only three of them (20, 23 and 24) remained active at 10 µM ( Figure 6B). The calculated Z´ values for both phenotype and motility of all schistosomula screens, related to this family of compound 7 analogues, are summarised in Supplementary Table 6 (see *Extended data* ^64^). Compounds 20, 23 and 24 were subsequently tested at lower concentrations to assess their anthelmintic potencies; EC _50_ values were derived from these dose response titrations ( Table 3).

These three compounds (20, 23 and 24) were next screened against adult worms at 12.50 µM (the sub-lethal concentration of parental compound 7) and all demonstrated greater potency than compound 7 in inhibiting motility and egg production at this concentration ( Figure 6C). These compounds were subsequently screened at a lower concentration (6.25 µM) aiming to discriminate the most potent compound amongst this third set of small molecules ( Figure 6D). This screen confirmed strong anthelmintic activities (motility and oviposition defects) for all compounds in comparison to the parental compound and indicated that compound 24 was the most potent.

### Miracidial transformation is inhibited by compound 7 and its structural analogues

**Figure 7.**
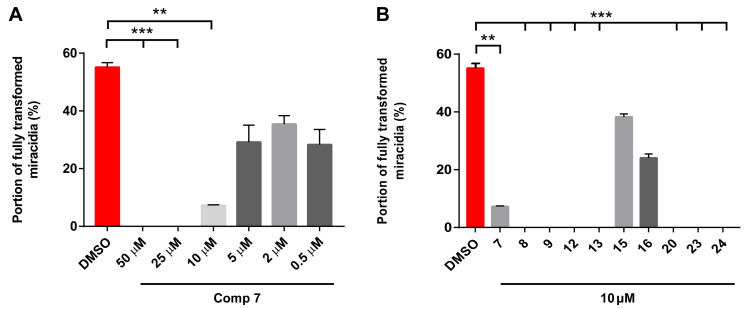
Compound 7 and its most active structural analogues block miracidia to primary sporocyst transformation. **Panel A** - The effect of compound 7 (50, 25, 10, 5, 2 and 0.5 μM) on miracidia transformation was registered in terms of % fully transformed sporocysts enumerated after 48 h. Each treatment was set up in triplicate and parasites were cultured in CBSS with 1% DMSO at a controlled temperature of 26°C (in the dark). **Panel B** - A drug screen was performed to assess the effect of compound 7 analogues at 10 μM to block miracidial transformation compared to the lead compound and DMSO (negative control). Statistical analysis was performed similarly to Figure 4. **, *** represent *p* < 0.0055, *p* < 0.0003, respectively.

To further assess the broader activities of these putative SmMLL-1 inhibitors on schistosome development, we next tested their ability to inhibit miracidia to sporocyst transformation ( Figure 7). Firstly, compound 7 demonstrated a clear dose-dependent effect on miracidia to sporocyst transformation; here, compound 7 was completely lethal to miracidia at 50 and 25 µM, whereas at 10 µM, compound 7 inhibited miracidia to sporocyst transformation by 45% ( Figure 7A). Inhibition of miracidia transformation declined to about 20–25% when the compound was tested at lower concentrations (5, 2 and 0.5 µM, Figure 7A). When the most active compound 7 analogues (compounds 8, 9, 12, 13, 15, 16, 20, 23 and 24) were next tested at 10 µM, all readily killed miracidia except for compounds 15 and 16 ( Figure 7B).

### Cytotoxicity of compound 7 and its structural analogues on HepG2 cells

Overt cytotoxicity was next explored on human HepG2 cells by prioritising those compounds that showed the most potent anti-schistosomal activities (EC _50_ on schistosomula equal or below 10 µM) starting from parent compound 7 and extending into its structural analogues. Each compound was tested in a dose response titration (200 to 0.01 µM) with the average CC _50_ of each compound reported in Table 3. Compound 20 was not toxic to this cell line, except for concentrations above 100 µM. In contrast, compounds 23 and 24 showed the highest toxicity (CC _50_ below 10 µM). All other compounds showed a range of moderate (10 µM < CC _50 _< 20 µM) to low (20 µM < CC _50 _< 60 µM) cytotoxicity on the selected cell line.

## Discussion

Careful regulation of histone methylation/demethylation is essential for developmental progression of the major human pathogen *S. mansoni* ^10, 24, 25, 36^. Therefore, exploration of the histone methylation machinery for druggable targets has been actively pursued ^10, 28, 29^. Here, we contribute to these studies by demonstrating that the *S. mansoni* histone methyltransferase Mixed Lineage Leukemia homologue (SmMLL-1, Smp_138030, Figure 1) is one such druggable epigenetic target.

The first evidence of SmMLL-1’s druggability came from functional genomics investigations of the transcript in adult schistosomes ( Figure 2). Here, partial siRNA-mediated knockdown of *smmll-1* (60%) resulted in adult worms physically detaching from the culture wells, moving significantly less and producing fewer eggs over a seven-day period post-treatment. While other phenotypic changes (e.g. coiled worms) did not accompany these siRNA-mediated effects, knockdown of the same target in a large-scale RNAi investigation confirmed a similar detachment phenotype for this target in adult schistosomes ^57^.

Consistent with our observations, functional genomics-led investigations have previously identified important roles for MLL homologues in other metazoans. In a variety of RNAi studies exploring *set-16* (MLL homologue) function in *C. elegans*, diverse phenotypes were observed including larval lethality ^68^, multivulva ^69^, disorganized oocytes ^70^, slow growth ^67^ and sterility ^71^. Overall, *set-16* was confirmed as an essential gene even though some reported RNAi phenotypes provide evidence that not all MLL deficiencies are lethal ^72^. Furthermore, distinct developmental defects have been observed in MLL1 ^-/-^ or MLL2 ^-/-^ mice whereas embryonic lethality resulted from MLL3, -4, and -5 deficiencies (observed in knockout mice) ^73, 74^. In the single cell eukaryote *Saccharomyces cerevisiae*, deletion of *set-1* (homologue of the human MLL-like protein) revealed a slow-growth defect ^75^. Collectively, these data suggest that H3K4 methylation (mediated by MLL homologues) has pleiotropic effects and the downstream phenotypic consequences of this epigenetic mark’s dis-regulation may depend on the organism in which it is studied. This explanation could justify our siRNA-mediated knockdown results highlighting that SmMLL-1 is not essential for schistosome viability, but is critical to other parasite features important for lifecycle maintenance, including motility (to retain position in the mesenteric venules of the definitive host ^57^) and fecundity. However, we cannot exclude that a more significant phenotype would be observed if a greater knockdown (above 60%) was achieved.

Based on the structural and functional characterisation of this target suggesting that SmMLL-1 (Smp_138030) was likely to function as a protein lysine methyltransferase (PKMT) with a currently unknown catalytic specificity, an antibody-based assay was used for measuring global histone H3K4 methylation in si *Smp_130830* treated worms. Here, only a slight decrease (9%) in global H3K4 methylation was observed in si *Smp_130830* treated worms compared to si *Luc* controls ( Figure 2E). As other related PKMTs are found in the *S. mansoni* genome (Smp_144180, Smp_070170 - closely related to MLL proteins - and Smp_140390 - closely related to the human SET1A/B) ^28, 76^, functional redundancy in the histone code could compensate for the minimal decrease in H3K4 methylation observed in si *Smmll-1* treated worms, similarly to what has been observed in *H. sapiens* ^77, 78^. Moreover, SmMLL-1’s main enzymatic substrate could be non-histone proteins found in the cytoplasm as has been recently shown for other PKMT homologues ^79– 81^. Therefore, a more detailed investigation of siRNA-mediated changes in SmMLL-1’s epitope (e.g. lysine methylation) on both nuclear and cytoplasm fraction derived from si *Smp_130830* treated parasites could be very informative in this regard. Nevertheless, it is clear from these functional genomics investigations that SmMLL-1 is involved in aspects of adult worm biology essential for mesenteric blood vessel positioning (attachment and coordinated movement) as well as lifecycle transmission (oviposition).

To progress the identification of small, drug-like molecules capable of binding to and inhibiting the activity of SmMLL-1, iterative structure-based virtual screening (SBVS) was applied. In the field of neglected tropical diseases, this approach has been widely used by multiple academic and industrial groups in drug discovery and is becoming an essential tool for assisting rapid and cost-efficient lead discovery ^82^. Successful applications of this approach for the discovery of novel anti-schistosomal agents include the identification of purine nucleoside phosphorylase-, tyrosine kinase-, 3-Oxoacyl-ACP reductase- and histone deacetylase- inhibitors ^83– 85^. For the work presented here on SmMLL-1, we focused on identifying compounds predicted to bind to the substrate-binding pocket over the cofactor-binding site since the former is usually less structurally conserved between functionally distinct proteins ^86^.

The putative inhibitors identified in this study were initially screened on schistosomula at 10 and 50 µM. The 10 µM concentration defines a cut-off value for the selection of hit compounds for further exploration in other lifecycle stages ^51^, whereas the 50 µM screen was included to avoid the loss of chemical information for exploring SAR and/or medicinal chemical optimisation. Compound 7 and its structural analogues (second and third set) shared the same 1,3,5-triazine (also called *s*-triazine) core linked to a piperazine ring (Supplementary Figure 4, *Extended data* ^64^), which interestingly was also present in the anti-bilharzial drug Bilharcid ^87^ and the protein kinase (PK) inhibitor Imatinib ^88^. The latter led to potent *in vitro* anti-schistosomal activity, which was lost *in vivo* likely due as a result of interaction with α-1-acid glycoprotein and serum albumin. Furthermore, a family of structural related derivatives (1,2,4-triazine) were also associated with anti-schistosomal activity ^89^, although limited information is currently available related to mechanism of action.

**Figure 8.**
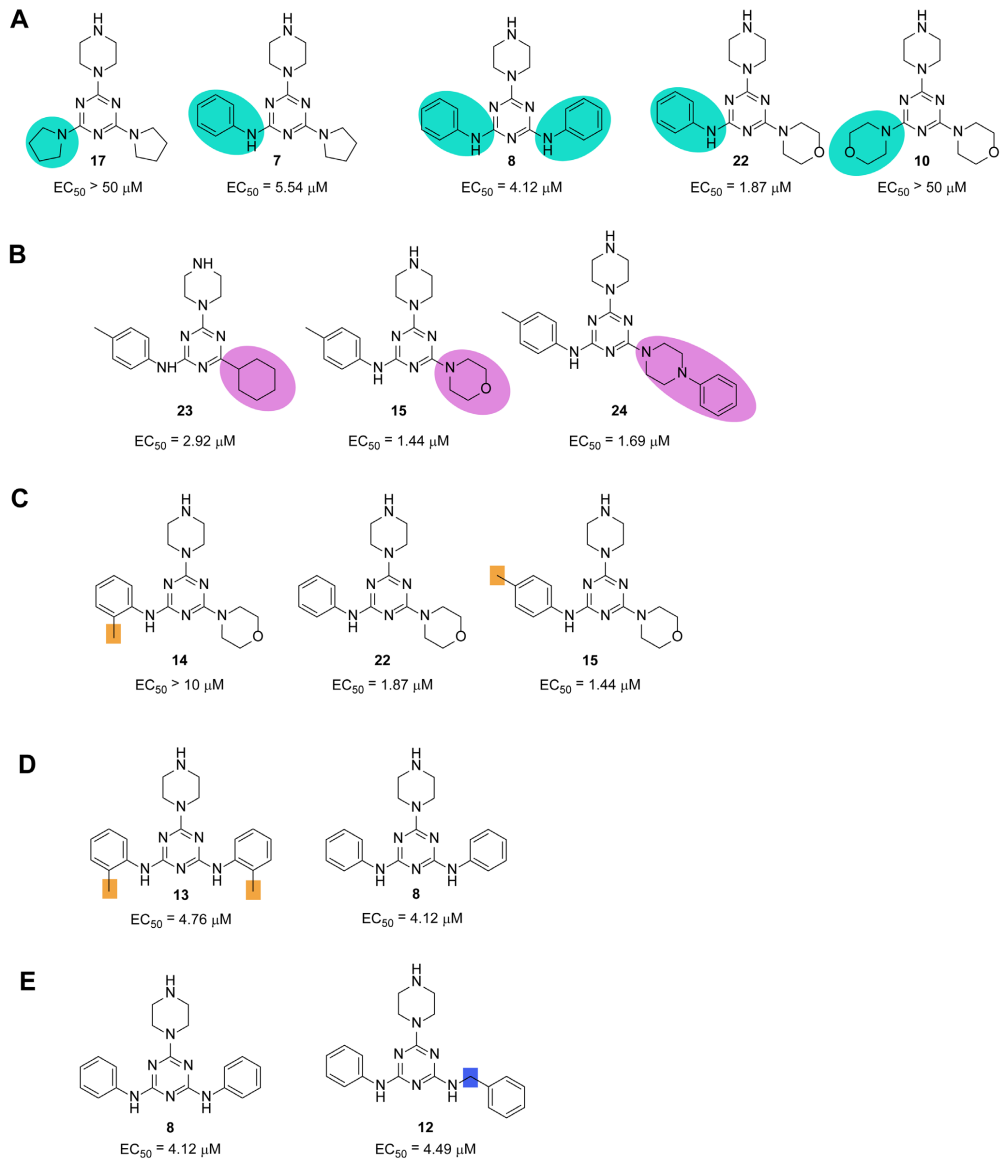
Structural activity relationship (SAR) of compound 7 analogues. Chemical structures of the compounds were analysed to investigate the effect of different chemical groups (corresponding to highlighted regions in cyan in **Panel A**, magenta in **Panel B**, orange in **Panel C** and **D**, blue in **Panel E**) on anti-schistosomula activity. EC _50_ values (derived from Table 3) were also reported for activity comparison.

The different patterns of piperazine ring substitution facilitated compound groupings (18 chemical entries in total) into two subfamilies: the monosubstituted-piperazines (N-substituted, Supplementary Figure 4A, *Extended data* ^64^) and the disubstituted-piperazines ((N,N’)-disubstituted piperazine, Supplementary Figure 4B, *Extended data* ^64^). The biological (schistosomes and HepG2 cells) screening of these compounds led to the definition of some preliminary SAR. Firstly, the presence of at least an aromatic ring linked to the triazine core generally increased the potency of the compounds (17 vs 7 and 10 vs 22, Figure 8A). However, compounds containing a double aromatic ring substitution did not seem to be as active as compounds containing a mixed ring substitution (one aromatic ring and one aliphatic ring (8 vs 7 and 8 vs 22). Moreover, the introduction of a spacer ( *N*-linker) between the triazine core and the aromatic ring negatively affected the activity of these chemicals (8 vs 12, Figure 8E).

**Figure 9.**
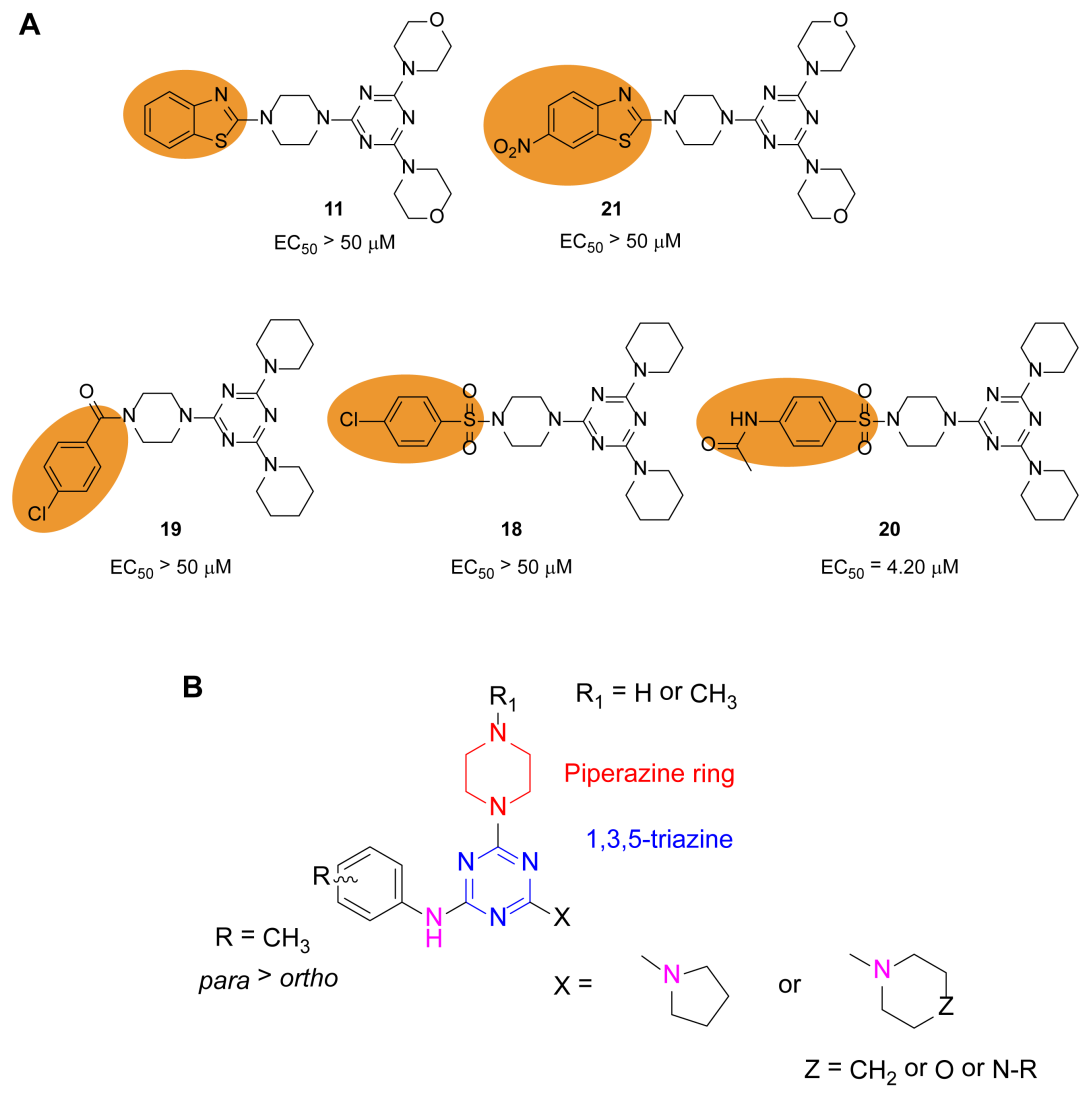
SAR of compound 7 analogues’ piperazine ring substitutions. **Panel A** - Chemical structures were analysed to investigate the effect of piperazine ring substitutions (corresponding to regions highlighted in orange) on schistosomula activity. **Panel B** - Summary of the SAR studies performed on the compounds presented in this study. A total of 18 structural analogues were used to generate this map. All biological results regarding the anti-schistosomal activity (EC _50_ values) were derived from Table 3.

Once the importance of an aromatic ring linked to the 1,3,5-triazine core was established, the correlation between anti-schistosomal activity and different ring substitutions in the second substitution was investigated. In summary, aliphatic rings containing heteroatoms (e.g. morpholine in 15 and piperazine in 24) were usually more active than the cyclohexyl ring (23, Figure 8B). The effect of alkyl substituents (a methyl group in this case, in orange Figure 8C and D) on the aromatic ring was investigated, leading to the conclusion that *ortho*-methyl substitution of the ring (14 vs 22 and 13 vs 8) reduced *in vitro* activity. However, the *para*-methyl substitution correlated with slightly improved activity (15). Moreover, compounds with a substitution of the piperazine ring (11, 21, 19 and 18, Figure 9A) showed activity only at high concentration (EC _50_ > 50 µM), with the exception being compound 20 (EC _50_ = 4.20 µM). Nevertheless, only a few compounds carrying a substitution on the piperazine ring were screened in this study, hence limited information is currently available to further inform and expand the preliminary SAR diagram of the compounds shown within this study ( Figure 9B). It is noteworthy that all compounds investigated in this study have a nitrogen in positions 2 and 4 of the triazine ring either included in a ring (e.g. substitution with pyrrolidine, morpholine and piperidine) or as linker (i.e. an aryl amino substitution shown in magenta in Figure 9B). This observation leaves space for further exploration of this structural feature, which would provide more insight on the pharmacophore of this family of anti-schistosomal compounds.

Docking simulations (Supplementary Figure 5, *Extended data* ^64^) to explore the putative binding mode of the central scaffold (6-(piperazin-1-yl)-1,3,5-triazine core) of these compounds to SmMLL-1’s substrate binding pocket confirmed a good fit (Supplementary Figure 5A, *Extended data* ^64^). Here, the triazine core consistently occupied the entrance of the lysine channel (Supplementary Figure 5B, *Extended data* ^64^) and engaged in arene-arene interactions with aromatic residues of the target (generally Phe1476 of Motif II of the SET domain, Supplementary Figure 5C, *Extended data* ^64^). The piperazine ring fitted in the lysine channels pointing towards the SAH cofactor and engaged with Ile1473 (Motif II) by a hydrogen bond. The other two rings linked to the triazine core occupied two additional pockets of the substrate binding pocket and adopted different conformations according to the different substitutions found for each compound.

The application of homology modelling and *in silico* virtual screening to this schistosome target resulted in the identification of novel anti-schistosomal compounds with a more favourable hit rate than what would be expected from a traditional high-throughput screening campaign ^90^. The selection criteria based on the compound docking score (used for the identification of the first and second set of chemicals) allowed us to address preliminary parasite-host selectivity considerations. In fact, the first (1–7) and second (8–16) set of compounds (Supplementary Table 1 and 3, respectively, see *Extended data* ^64^) all showed potent anti-schistosomal activity (EC _50_ < 10 µM) and moderate to low cytotoxicity on HepG2 cells (CC _50_ > 10 µM, Table 3). Amongst the final set of compounds (17–24, Supplementary Table 5, *Extended data* ^64^), two of the four hits at 10 µM (compounds 24 and 23) showed the highest toxicity; this could be explained by a higher affinity of these chemicals to the human homologue (HsMLL3) over SmMLL-1, which was an exclusion factor used during the initial selection of compounds 1-16. However, a docking score-based approach has some limitations as we would have missed information on active compounds 20, 22, 23 and 24 with EC _50_ < 10 µM. To overcome this, we applied an integrative methodology in this study. Firstly, compounds were selected based on docking simulation scores (difference in schistosome vs host target binding) and, secondly, extended to the exploration of chemical space around the central anti-schistosomal hit scaffold. We felt this approach provided the most effective way of identifying putative SmMLL-1 inhibitors containing a 1,3,5-triazine core with a piperazine ring regardless of host cytotoxicity or docking software limitations (e.g. approximated scoring functions, which often provide computational results that do not correlate with experimental readouts) ^91, 92^.

With broad activity against miracidia, schistosomula and adults (aligned to *smmll-1*/ *smp_138030*’s transcript abundance throughout the schistosome lifecycle ^28^), this compound class was equally effective in halting key transitions in schistosome development ( Figure 3, Figure 4, Figure 5, Figure 6 and Figure 7). A notable activity against adult worms was observed since both motility and egg production were both significantly impaired ( Figure 4, Figure 5 and Figure 6). These defects recapitulated the *smmll-1* knockdown phenotypes ( Figure 2) and further validated SmMLL-1 as a molecular target of this compound class. However, we cannot rule out the possibility that the anti-parasitic activities of these compounds, at least in part, may have been due to inhibition of other SmHMTs or completely different targets within the parasite. As damage to the adult surface was observed during sub-lethal compound treatment (e.g. compound 7; Supplementary Figure 3, *Extended data* ^64^), but not observed during si *smmll-1* RNAi studies, this hypothesis is currently under investigation.

In summary, using a combination of drug-discovery approaches, we have identified a novel chemical scaffold (the 6-(piperazin-1-yl)-1,3,5-triazine) predicted to bind to SmMLL-1’s substrate binding pocket. Central features associated with this scaffold’s anti-schistosomal potency include both a triazine ring usually substituted with a combination of an aliphatic ring (or a heterocycle) and an aromatic ring (with a *para* rather than an *ortho* substitution). Further medicinal chemistry optimisation should be advanced to identify other aromatic ring/piperazine ring substitutions or different linkers connecting the triazine core to other portions of the molecule. By doing so, the progression of a chemical scaffold, not previously associated with anti-schistosomal activity, could lead to the development of a new therapeutic targeting a critical epigenetic enzyme.

## Data availability

### Underlying data

Figshare: Identification of 6-(piperazin-1-yl)-1,3,5-triazine as a chemical scaffold with broad anti-schistosomal activities – Underlying data. https://doi.org/10.6084/m9.figshare.12473108.v4 ^93^.

This project contains the following underlying data:

- 1. RNAi-mediated knockdown of smp_138030/smmll-1.xlsx
- 2. Schistosomula drug screen of putative SmMLL-1 inhibitors.xlsx
- 3. Adult worm drug screen of putative SmMLL-1 inhibitors.xlsx
- 4. Anti-schistosomal activity of compound 7 structural analogues (set two).xlsx
- 5. Anti-schistosomula activity of the remaining compound 7 analogues.xlsx
- 6. Miracidia drug screen.xlsx
- 7. Raw data for EC50 and CC50 calculation.xlsx
- 8. Schistosomula titration of compound 7.xlsx
- 9. SEM images.pptx
- 10. Somula microscopy images (related to Fig 3).pptx
- 11. Somula microscopy images (Supp Fig 2).pptx

Data are available under the terms of the Creative Commons Attribution 4.0 International license (CC-BY 4.0).

### Extended data

Figshare: Identification of 6-(piperazin-1-yl)-1,3,5-triazine as a chemical scaffold with broad anti-schistosomal activities – Extended data. https://doi.org/10.6084/m9.figshare.12546449.v2 ^64^.

This project contains the following extended data:

- Supplementary Table 1 (List of putative SmMLL-1 inhibitors (first set) identified by structure-based virtual screening)
- Supplementary Table 2 (Phenotype and motility Z´ values for both primary (single-point concentration) and secondary (titration) screens of compound set one)
- Supplementary Table 3 (List of compound 7 structural analogues (second set) identified as putative SmMLL-1 inhibitors by structure-based virtual screening)
- Supplementary Table 4 (Phenotype and motility Z´ values for both primary (single-point concentration) and secondary (titration) screens of compound set two)
- Supplementary Table 5 (List of remaining compound 7 analogues available in the Specs fragment-based library)
- Supplementary Table 6 (Phenotype and motility Z´ values for both primary (single-point concentration) and secondary (titration) screens of remaining compound 7 analogues available in the Specs fragment-based library)
- Supplementary Figure 1 (The homology model of SmMLL-1/Smp_138030 is of high quality according to three different metrics)
- Supplementary Figure 2 (Compound 7 demonstrates moderate anti-schistosomula potency)
- Supplementary Figure 3 (A sub-lethal concentration of compound 7 induces surface and tegumental alterations in adult schistosomes)
- Supplementary Figure 4 (Chemical structure and anti-schistosomal properties (EC _50_s) of the 18 compounds investigated in this study)
- Supplementary Figure 5 (Hypothetical binding mode of the compound 7 analogues to the predicted target SmMLL-1)

Data are available under the terms of the Creative Commons Attribution 4.0 International license (CC-BY 4.0).

## Software availability

Source code available from: https://bitbucket.org/gildagilda/git_wormassaygp2

Archived source code at time of publication: https://doi.org/10.5281/zenodo.3929417 ^55^.

License: Creative Commons Attribution 4.0 International license (CC-BY 4.0).
