# Supplementary material for: Identification of 6-(piperazin-1-yl)-1,3,5-triazine as a chemical scaffold with broad anti-schistosomal activities
Source: Wellcome Open Res. 2020 Nov 13;5:169. Originally published 2020 Jul 17. [Version 2] doi: 10.12688/wellcomeopenres.16069.2 (PMC7459852; doi:10.12688/wellcomeopenres.16069.2)
Supplement: Peer review history

# Peer review: Reviewer response for version 2

**DOI:** 10.21956/wellcomeopenres.18029.r41424

**Published:** 2020-12-23

**Authors:** Giovina Ruberti

**Affiliations:** 1 National Research Council, Institute of Biochemistry and Cell Biology, Rome, Italy

The authors have satisfactorily responded to all my questions.

Is the work clearly and accurately presented and does it cite the current literature?

Yes

If applicable, is the statistical analysis and its interpretation appropriate?

Yes

Are all the source data underlying the results available to ensure full reproducibility?

Yes

Is the study design appropriate and is the work technically sound?

Yes

Are the conclusions drawn adequately supported by the results?

Partly

Are sufficient details of methods and analysis provided to allow replication by others?

Yes

Reviewer Expertise:

NA

I confirm that I have read this submission and believe that I have an appropriate level of expertise to confirm that it is of an acceptable scientific standard.

# Peer review: Reviewer response for version 2

**DOI:** 10.21956/wellcomeopenres.18029.r41422

**Published:** 2020-11-24

**Authors:** David Williams

**Affiliations:** 1 Department of Microbial Pathogens and Immunity, Rush University Medical Center, Chicago, IL, USA

The authors addressed all of my comments on the version reviewed.

Is the work clearly and accurately presented and does it cite the current literature?

Yes

If applicable, is the statistical analysis and its interpretation appropriate?

Yes

Are all the source data underlying the results available to ensure full reproducibility?

Yes

Is the study design appropriate and is the work technically sound?

Yes

Are the conclusions drawn adequately supported by the results?

Partly

Are sufficient details of methods and analysis provided to allow replication by others?

Yes

Reviewer Expertise:

Molecular parasitology, host-parasite interactions, drug development.

I confirm that I have read this submission and believe that I have an appropriate level of expertise to confirm that it is of an acceptable scientific standard.

# Peer review: Reviewer response for version 2

**DOI:** 10.21956/wellcomeopenres.18029.r41423

**Published:** 2020-11-16

**Authors:** Adriana Erica Miele

**Affiliations:** 1 Institute of Chemistry, Molecular and Supramolecular Biochemistry(ICBMS), UMR 5246 CNRS - UCBL, University of Lyon, Lyon, France; 2 Dept. Biochemical Sciences, Sapienza University of Rome, Rome, Italy

The authors have replied to the comments and modified the manuscript accordingly.

Is the work clearly and accurately presented and does it cite the current literature?

Yes

If applicable, is the statistical analysis and its interpretation appropriate?

Yes

Are all the source data underlying the results available to ensure full reproducibility?

Yes

Is the study design appropriate and is the work technically sound?

Yes

Are the conclusions drawn adequately supported by the results?

Yes

Are sufficient details of methods and analysis provided to allow replication by others?

Yes

Reviewer Expertise:

Molecular parasitology, host-parasite interactions, structural biochemistry.

I confirm that I have read this submission and believe that I have an appropriate level of expertise to confirm that it is of an acceptable scientific standard.

# Peer review: Reviewer response for version 1

**DOI:** 10.21956/wellcomeopenres.17631.r39614

**Published:** 2020-08-28

**Authors:** Giovina Ruberti

**Affiliations:** 1 National Research Council, Institute of Biochemistry and Cell Biology, Rome, Italy

Padalino *et al*. used a multidisciplinary approach (functional genomics, molecular modeling and efficacy studies on different developmental stages of *S. mansoni*) for the discovery of novel targets and drugs for the treatment of schistosomiasis. Schistosomiasis is a neglected tropical parasitic disease affecting more than 200 million people in the poorest regions of the world. Today, the treatment and most control initiatives for schistosomiasis rely on a single drug, praziquantel (PZQ) that has high efficacy, few and transient side effects, competitive cost. However, while very active on adult parasites, PZQ is poorly effective against juvenile worms both *in vitro* and *in vivo*. Moreover, the constant and massive use of a single drug has raised concerns about the possibility of emerging drug resistance. Therefore, the discovery of new drug targets and compounds is required. Targeting the epigenome has emerged as a new strategy for the treatment of parasitic diseases including schistosomiasis.

 The present study identifies a novel potentially relevant drug target, a histone methyl transferase (HMT), SmMML-1 in *Schistosoma mansoni*. Moreover, new multi-stage active compounds were identified and characterized.

 Overall the work is well written, the study is well hypothesized, designed and executed. The results support the majority of the conclusions. Unfortunately, it is not clear at this stage whether the novel compounds are acting on the MLL-1 homologue in *S. mansoni*. I think the following suggestions could help you to address this open issue.

- Smp_138030 was characterized as the closest homologue of the human mixed lineage leukemia (MLL) HMT. Silencing of Smp_138030 (60%) resulted in motility and egg production defects in adult worms; however, no statistically significant change of H3K4 methylation was detected in knockdown parasites. This finding suggests that the MLL-1 homologue in *S. mansoni* might target different enzymatic substrates and/or that silencing of other HMT genes is required to impair H3K4 methylation in *S. mansoni*.
  Gene expression studies of siLuc and siSmp_138030 parasites could lead to the identification of a SmMLL-1-associated signature. In turn this signature could be compared with the one derived from compound 7 and compound 7 analogs-treated parasites in order to support the hypothesis that the novel compounds are targeting SmMLL-1;
- Viability assays and phenotyping studies could be performed in siLuc and siSmp_138030 parasites treated with sub-lethal or lethal doses of compound 7. Differences in the gene-silenced compound-treated parasites could support the hypothesis that the novel schistosomicidal compound is targeting SmMLL-1;
- H3K4 methylation should be also investigated in parasites treated with compound 7 and/or its structural analogues;
- H3K4 methylation could be investigated in mammalian cells treated with compound 7 and/or its structural analogues in order to support the HMT inhibitory activity of the novel compounds.

Is the work clearly and accurately presented and does it cite the current literature?

Yes

If applicable, is the statistical analysis and its interpretation appropriate?

Yes

Are all the source data underlying the results available to ensure full reproducibility?

Yes

Is the study design appropriate and is the work technically sound?

Yes

Are the conclusions drawn adequately supported by the results?

Partly

Are sufficient details of methods and analysis provided to allow replication by others?

Yes

Reviewer Expertise:

Biology of Schistosoma mansoni, drugs and targets discovery.

I confirm that I have read this submission and believe that I have an appropriate level of expertise to confirm that it is of an acceptable scientific standard, however I have significant reservations, as outlined above.

# Response

**Published:** 2020-10-15

**Authors:** Gilda Padalino

**Affiliations:** Aberystwyth University, UK

**We thank the Reviewer very much for the detailed and constructive comments on our manuscript entitled: “Identification of 6-(piperazin-1-yl)-1,3,5-triazine as a chemical scaffold with broad anti-schistosomal activities”, submitted to *Wellcome Open Research*.**

Reviewer #3 Comments:

Padalino et al. used a multidisciplinary approach (functional genomics, molecular modeling and efficacy studies on different developmental stages of S. mansoni) for the discovery of novel targets and drugs for the treatment of schistosomiasis. Schistosomiasis is a neglected tropical parasitic disease affecting more than 200 million people in the poorest regions of the world. Today, the treatment and most control initiatives for schistosomiasis rely on a single drug, praziquantel (PZQ) that has high efficacy, few and transient side effects, competitive cost. However, while very active on adult parasites, PZQ is poorly effective against juvenile worms both in vitro and in vivo. Moreover, the constant and massive use of a single drug has raised concerns about the possibility of emerging drug resistance. Therefore, the discovery of new drug targets and compounds is required. Targeting the epigenome has emerged as a new strategy for the treatment of parasitic diseases including schistosomiasis.

The present study identifies a novel potentially relevant drug target, a histone methyl transferase (HMT), SmMML-1 in Schistosoma mansoni. Moreover, new multi-stage active compounds were identified and characterized.

**Comment: **Overall the work is well written, the study is well hypothesized, designed and executed. The results support the majority of the conclusions. Unfortunately, it is not clear at this stage whether the novel compounds are acting on the MLL-1 homologue in S. mansoni. I think the following suggestions could help you to address this open issue.

 

- Smp_138030 was characterized as the closest homologue of the human mixed lineage leukemia (MLL) HMT. Silencing of Smp_138030 (60%) resulted in motility and egg production defects in adult worms; however, no statistically significant change of H3K4 methylation was detected in knockdown parasites. This finding suggests that the MLL-1 homologue in S. mansoni might target different enzymatic substrates and/or that silencing of other HMT genes is required to impair H3K4 methylation in S. mansoni.

 

**Response:** Please see our response to Reviewer 2’s almost identical query.

Bioinformatic characterisation of *S. mansoni* genome identified three different genes encoding schistosome proteins containing MLL-specific domains (Smp_138030, Smp_144180 and Smp_070170) (Ref: doi.org/10.1016/j.ijpddr.2018.10.005).

The human MLL family includes five protein members, which are involved in mono-, di-, and trimethylation of H3K4 (Ref: doi.org/10.17554/j.issn.2313-5611.2015.01.17). However, the catalytic specificity of each human MLL homologue is currently unclear (Ref: doi.org/10.7554/eLife.01503.001). Similarly, we can’t exclude a functional redundancy in the histone code since the same epitope (e.g. the methylation of a specific amino acid residue) can be introduced by several components of the histone methylation machinery in the parasite.

Thus, it can be concluded that methylation activity in siSmp_138030 treated worms (60% knockdown) can be compensated for by other components of the schistosome methylation machinery (such as Smp_070170 and Smp_144180) leading to only a slight demethylation effect (9% decrease in global H3K4 methylation).  We have postulated this in our discussion.

 We also can’t exclude that siRNA-mediated silencing of the other two members of the MLL family (individually or in combination with Smp_138030) would result in a stronger phenotype. An ongoing project is looking into this by assessing the phenotypes of worms treated with siRNAs directed against all three MLL homologs.  

- Gene expression studies of siLuc and siSmp_138030 parasites could lead to the identification of a SmMLL-1-associated signature. In turn this signature could be compared with the one derived from compound 7 and compound 7 analogs-treated parasites in order to support the hypothesis that the novel compounds are targeting SmMLL-1;

    

- Viability assays and phenotyping studies could be performed in siLuc and siSmp_138030 parasites treated with sub-lethal or lethal doses of compound 7. Differences in the gene-silenced compound-treated parasites could support the hypothesis that the novel schistosomicidal compound is targeting SmMLL-1;

    

- H3K4 methylation should be also investigated in parasites treated with compound 7 and/or its structural analogues;

 

**Response:** We understand the importance of investigating the methylation status of inhibitor-treated worms to likely confirm the mechanism of action of these compounds.

Therefore, we are planning to perform this additional experiment with compound 7 and/or one of the most active compounds presented in this study. Once we have collected experimental data in this regard, we’ll include them in an new version of the manuscript.

- H3K4 methylation could be investigated in mammalian cells treated with compound 7 and/or its structural analogues in order to support the HMT inhibitory activity of the novel compounds.

  

We would like to thank the Reviewer and the Editorial Team very much for their detailed and very constructive comments, which have helped us to improve our manuscript. We hope that this point-by-point response could clarify the open questions and that our suggested revisions are clear and convincing. We are, of course, happy to discuss any further open issues.

Kind regards,

Gilda Padalino (on behalf of the authors)

# Peer review: Reviewer response for version 1

**DOI:** 10.21956/wellcomeopenres.17631.r39616

**Published:** 2020-08-17

**Authors:** David Williams

**Affiliations:** 1 Department of Microbial Pathogens and Immunity, Rush University Medical Center, Chicago, IL, USA

This study focuses on drug discovery for schistosomiasis, a neglected tropical disease affecting 200,000,000 people. Treatment options are limited to a single drug, which has poor activity against some stages of worm development. The evolution of drug resistance is highly likely, with no alternative compounds for treatment. The topic is of great value to human health.

 Recent efforts to identify new drugs for schistosomiasis have identified epigenetic targets. The present study identifies and characterizes a new epigenetic target, a histone methyl transferase in *Schistosoma mansoni*. Functional genomic approaches were used to validate druggability of the target. Molecular modeling was used to identify potential compounds binding to the protein. Selected compounds were purchased and tested for anti-schistosome activities. Results guided the selection of analogs for further studies. The paper is well written and the data is clearly presented. The results support the conclusions for the most part.

 Determination of specificity of action (Table 3) is not based on an equivalent analysis of activity in schistosomes and mammalian cells; different end-points were measured and a comparison is not useful. Worms and somula were incubated for 72 hr with compound, then phenotypic analysis was done. HepG2 cells were cultured for either 24 hr or 20 hr (which one?) with compound, then viability assessed by MTT. Since different parameters are measured it is not possible to conclude that the inhibitors are worm selective.

 Assay quantifying methylation on histone H3, lysine 4 (H3K4) was performed on adult worm histone protein extracts. These assays revealed that smp_138030 knockdown resulted in a not statistically significant change of H3K4 methylation. A 60% reduction in mRNA abundance resulted in no change in methylation. Was methylation status of inhibitor-treated worms assessed? This would show that compound anti-worm activity was through inhibition of SmMLL-1 and not off target effects. Evidence that the compounds function through inhibition of SmMLL-1 is lacking.

 How many MLL paralogues are present in schistosomes? What is their identity? If this family of proteins is targeted, do all need to be silenced/inhibited to see a strong phenotype?

 A discussion of known MLL-1 inhibitors with comparison to the new inhibitors identified would be useful. Why were known MLL-1 inhibitors not investigated?

Is the work clearly and accurately presented and does it cite the current literature?

Yes

If applicable, is the statistical analysis and its interpretation appropriate?

Yes

Are all the source data underlying the results available to ensure full reproducibility?

Yes

Is the study design appropriate and is the work technically sound?

Yes

Are the conclusions drawn adequately supported by the results?

Partly

Are sufficient details of methods and analysis provided to allow replication by others?

Yes

Reviewer Expertise:

Molecular parasitology, host-parasite interactions, drug development.

I confirm that I have read this submission and believe that I have an appropriate level of expertise to confirm that it is of an acceptable scientific standard, however I have significant reservations, as outlined above.

# Response

**Published:** 2020-10-15

**Authors:** Gilda Padalino

**Affiliations:** Aberystwyth University, UK

**We thank the Reviewer very much for the detailed and constructive comments on our manuscript entitled: “Identification of 6-(piperazin-1-yl)-1,3,5-triazine as a chemical scaffold with broad anti-schistosomal activities”, submitted to *Wellcome Open Research*.**

Reviewer #2 Comments:

 This study focuses on drug discovery for schistosomiasis, a neglected tropical disease affecting 200,000,000 people. Treatment options are limited to a single drug, which has poor activity against some stages of worm development. The evolution of drug resistance is highly likely, with no alternative compounds for treatment. The topic is of great value to human health.

 Recent efforts to identify new drugs for schistosomiasis have identified epigenetic targets. The present study identifies and characterizes a new epigenetic target, a histone methyl transferase in Schistosoma mansoni. Functional genomic approaches were used to validate druggability of the target. Molecular modeling was used to identify potential compounds binding to the protein. Selected compounds were purchased and tested for anti-schistosome activities. Results guided the selection of analogs for further studies. The paper is well written and the data is clearly presented. The results support the conclusions for the most part.

**Comment: **Determination of specificity of action (Table 3) is not based on an equivalent analysis of activity in schistosomes and mammalian cells; different end-points were measured and a comparison is not useful. Worms and somula were incubated for 72 hr with compound, then phenotypic analysis was done. HepG2 cells were cultured for either 24 hr or 20 hr (which one?) with compound, then viability assessed by MTT. Since different parameters are measured it is not possible to conclude that the inhibitors are worm selective.

**Response:** Cells were incubated for 20 h with the compound prior application of MTT reagent. After 4 h incubation (24 h drug-cell incubation in total), the absorbance reading at 570 nm was measured with a POLARstar Omega. The methodology has been updated accordingly.

 Our rationale for choosing a 24 h window for HepG2 cytotoxicity estimations is based on two reasons. The first reason originates from the literature ( Ref: doi.org/10.1371/journal.pone.0177902). Here, as part of the U.S. Tox21 screening program, HepG2 cytotoxicity of 10K chemicals was investigated at 0, 8, 16, 24, 32, and 40 h of exposure in a concentration dependent fashion. The results of these screens indicated that maximal HepG2 cytotoxicity (measuring metabolic activity, which is how we measured cytotoxicity in our study) of active compounds was found before 24 hours in 91% of the assays. This large-scale cytotoxicity screen indicated to us that an appropriate window for testing this library of compounds on a uniform monolayer of HepG2 cells was 24 (O/N) h. We have now included this reference in the Methods section to provide further justification of the 24 h interval. 

 The second reason relates to the nature of the biological material we are comparatively testing in our *in vitro* assays. In comparison to a monolayer of HepG2 cells, schistosomes (schistosomula and adult worms) are much larger, multi-cellular, multi-tissue organisms covered by a heptalaminate membrane. We reasoned that compound-induced anthelmintic activities would take longer to develop in these metazoan organisms when compared to the HepG2 cells. This is the rationale for quantifying anti-schistosome phenotype and motility metrics at 72 h. 

 Above all, the point raised by the reviewer is understandable, so we agreed to remove the selectivity index column from Table 3 and believe this won’t affect the major findings reported in the manuscript. We also amended the results section related to the cytotoxicity.

**Comment: **Assay quantifying methylation on histone H3, lysine 4 (H3K4) was performed on adult worm histone protein extracts. These assays revealed that smp_138030 knockdown resulted in a not statistically significant change of H3K4 methylation. A 60% reduction in mRNA abundance resulted in no change in methylation. Was methylation status of inhibitor-treated worms assessed? This would show that compound anti-worm activity was through inhibition of SmMLL-1 and not off target effects. Evidence that the compounds function through inhibition of SmMLL-1 is lacking.

**Response:** This comment correctly highlights how the investigation of the methylation status of inhibitor-treated worms could confirm the mechanism of action of these compounds.

 We haven’t planned this experiment because we wanted firstly to see if a greater knockdown of Smp_138030 would result in a more significant change of H3K4 methylation and secondly assess the presence of a compensatory effect caused by other members of the MLL family (see comment below).

 Nevertheless, we are planning to perform this additional experiment with compound 7 and/or one of the most active compounds presented in this study. The results will then be included in a new version of the manuscript.

**Comment: **How many MLL paralogues are present in schistosomes? What is their identity? If this family of proteins is targeted, do all need to be silenced/inhibited to see a strong phenotype?

**Response: **Bioinformatic characterisation of *S. mansoni* genome identified three different genes encoding schistosome proteins containing MLL-specific domains (Smp_138030, Smp_144180 and Smp_070170) (Ref: doi.org/10.1016/j.ijpddr.2018.10.005).

 The human MLL family includes five protein members, which are involved in mono-, di-, and trimethylation of H3K4 (Ref: doi.org/10.17554/j.issn.2313-5611.2015.01.17). However, the catalytic specificity of each human MLL homologue is currently unclear (Ref: doi.org/10.7554/eLife.01503.001). Similarly, we can’t exclude a functional redundancy in the histone code since the same epitope (e.g. the methylation of a specific amino acid residue) can be introduced by several components of the histone methylation machinery in the parasite.

 Thus, it can be concluded that methylation activity in siSmp_138030 treated worms (60% knockdown) can be compensated for by other components of the schistosome methylation machinery (such as Smp_070170 and Smp_144180) leading to only a slight demethylation effect (9% decrease in global H3K4 methylation).  We have postulated this in our discussion.

 We also can’t exclude that siRNA-mediated silencing of the other two members of the MLL family (individually or in combination with Smp_138030) would result in a stronger phenotype. An ongoing project is looking into this by assessing the phenotypes of worms treated with siRNAs directed against all three MLL homologs.  

   

**Comment: **A discussion of known MLL-1 inhibitors with comparison to the new inhibitors identified would be useful. Why were known MLL-1 inhibitors not investigated?

**Response: **Pharmacological targeting of HsMLL substrate or cofactor binding pockets has so far been unsuccessful (Ref: doi.org/10.1002/pro.3129). The main drug discovery efforts around this target have focused on small molecules disrupting chromatin complexes (Ref: doi.org/10.1016/j.chembiol.2016.07.014 and doi.org/10.1002/pro.3129). For instance, HsMLL binds via its N terminus to Menin and via its C terminus to WDR5, two molecular scaffolds coordinating the functions of multiple proteins.

 We have investigated the activity of two commercially available chemical probes: MI-1 (SML0537, Sigma) and OICR-9429 (kindly provided by Structure Genomics Consortium - https://www.thesgc.org/chemical-probes/OICR-9429). The first probe targets the MLL-binding domain of Menin and effectively disrupts the Menin-MLL interaction (Ref: doi.org/10.1038/nchembio.773); the second targets the MLL-binding domain of WDR5, disrupting the WDR5-MLL interaction (Ref: doi.org/10.1038/nchembio.1859).

 These two compounds were screened on schistosomula at 10 µM (Figure A) and only MI-1 was identified as hit. A subsequent titration of this compound (Figure B) didn’t show a strong anti-schistosomal activity (EC _50 _of 3.97 and 2.03 µM for phenotype and motility, respectively – Figure B).

Figure A - Mechanically-transformed schistosomula (n = 120) were incubated with the selected compounds (10 µM of MI-1 and OICR-9429 in 0.625% DMSO; each of them in duplicate) for 72 h at 37°C in a humidified atmosphere containing 5% CO _2_. Three independent screens (each including two technical replicates) were performed for each compound. Phenotype and motility Z´ values for each screen are summarized in the following table. Compounds with activity on both schistosomula phenotype and motility are shown within the hit zone (delineated by the dotted red lines in the graph).

Figure B - Dose-response titration (10, 5, 2.5, 1.25 and 0.625 µM) of MI-1 on mechanically-transformed schistosomula looking at both phenotype (P) and motility (M) scores. Three independent dose response titrations were performed, and each compound concentration was evaluated in duplicate. One of three replicate screens is shown here (barcode 0359). The dotted red line identifies the hit zone.

 We haven’t included these results in the manuscript because their mechanism of action is quite different to the putative mechanism of action of molecules described in this study.  Therefore, their different anti-schistosomal results cannot be directly compared.

 We would like to thank the Reviewer and the Editorial Team very much for their detailed and very constructive comments, which have helped us to improve our manuscript. We hope that this point-by-point response could clarify the open questions and that our suggested revisions are clear and convincing. We are, of course, happy to discuss any further open issues.

 Kind regards,

 Gilda Padalino (on behalf of the authors)

# Peer review: Reviewer response for version 1

**DOI:** 10.21956/wellcomeopenres.17631.r39615

**Published:** 2020-07-23

**Authors:** Adriana Erica Miele

**Affiliations:** 1 Institute of Chemistry, Molecular and Supramolecular Biochemistry(ICBMS), UMR 5246 CNRS - UCBL, University of Lyon, Lyon, France; 2 Dept. Biochemical Sciences, Sapienza University of Rome, Rome, Italy

The manuscript by Padalino and co-Authors presents a complete process of lead discovery, from target validation to virtual screening, from high content screening against different life stages of *Schisotoma mansoni* parasites to preliminary SAR and selectivity tests to guide in lead choice and optimization. The results on the new scaffold, based on a piperazine ring connected to a 1,3,5-triazine core, are indeed very promising. The optimised leads built from compound 7 represent good expectations in the quest for new drugs against schistosomiasis.

 All the data are very well explained and all the methods are clearly presented, either in the manuscript or in the numerous supplementary files.

 One curiosity on the bioinformatics tools/software used: it is true that many programmes nowadays are available to perform both homology modelling and ligand docking, but which is the point to give only one alternative in the manuscript? I refer to the sentence at p.4 paragraph 3 "Modeller could be a valid alternative"; a similar sentence at p. 5 paragraphs 3 and 4 and in Figure 1 "AutoDock could alternatively be used .."

 If Authors have used only one programme (MOE and Glide) for each purpose and for any good reason, why give only one alternative? There are many others out there.

 On the other hand, if the Authors have used both programmes (MOE and Modeller; Glide and AutoDock), then it could be wise to give a comparison of the results based on the two softwares.

 The rationale of the research project is clear and the manuscript reads easily, without the need to go back and forth to the supplementary files, unless for an in depth examination and thorough understanding.

 A few minor comments/suggestions:

- In the keywords Authors might add MLL, unless there is a limit given in the journal's instructions;
- p. 4 column 1 line 1: there is a typo in  " *ad libitum*";
- p. 4 column 1 lines 7 and 11: the buffer used is PBS. 1x does not mean anything, unless you give the recipe
- p. 4 column 1, paragraph 2 line 1: "double saline solution" instead of 2x saline;
- p. 4 column 1 "Homology modelling". Does 58% sequence similarity between human and schistosoma MLL refer to the SET domain alone? In that case, please use both PDB ID and chain ID (here 5F6K:C);
- p. 5 SBVS framework: why have the Authors used the OPLS_2005 force field instead of the newest OPLS3?
- Figure 1 panel C legend: *“Same orientation as in B, but highlighting the structural motifs I to IV in black.”* instead of “Schematic representation of Smp_138030’s domain architecture with indication of the different motifs (black regions on the protein represented as green ribbon)”.
- The yellow ribbon is “peptide form histone H3”, not “histone protein”.
- Figure 2 legend: a space is missing between “indicated.All siRNA”
- Figure 2 *vs* Fig. 4,5,7: why the p-values of figure 2 are different from those of the other figures? Are the cut-off different? Moreover the ** value is missing in figure 2.
- Figure 4B: for clarity it would be better to present the data from 0 to 50 and then a broken y axis line and start again from 300 to 400, so that the tiny differences between PZQ and compound 7 could be highlighted.
- Figure 5 legend: were the measurements done in duplicate or in triplicate?
- Table 3: SI for compound 20 is >24, not ND
- Figure 6: the title of this figure should be the same as that of Figure 5 with the exception of Set 3. The present title is misleading → it cites schistosomula and then presents the egg counts.
- Figure 6C and 6D *vs* 4B and 5D: why are the DMSO egg counts at 150 (6C,D) instead of 300 (4B,5D)?
- Figure 7: p-values are missing in the legend.
- p. 13 column 2 “Discussion”: please use “Therefore” instead of “Because of this realisation”
- p. 14 column 2 line 4: insert a “,” between “maintenance” and “including”

Is the work clearly and accurately presented and does it cite the current literature?

Yes

If applicable, is the statistical analysis and its interpretation appropriate?

Yes

Are all the source data underlying the results available to ensure full reproducibility?

Yes

Is the study design appropriate and is the work technically sound?

Yes

Are the conclusions drawn adequately supported by the results?

Yes

Are sufficient details of methods and analysis provided to allow replication by others?

Yes

Reviewer Expertise:

Molecular parasitology, host-parasite interactions, structural biochemistry

I confirm that I have read this submission and believe that I have an appropriate level of expertise to confirm that it is of an acceptable scientific standard.

# Response

**Published:** 2020-10-15

**Authors:** Gilda Padalino

**Affiliations:** Aberystwyth University, UK

**We thank the Reviewer very much for the detailed and constructive comments on our manuscript entitled: “Identification of 6-(piperazin-1-yl)-1,3,5-triazine as a chemical scaffold with broad anti-schistosomal activities”, submitted to *Wellcome Open Research*.**

Reviewer #1 Comments:

The manuscript by Padalino and co-Authors presents a complete process of lead discovery, from target validation to virtual screening, from high content screening against different life stages of Schisotoma mansoni parasites to preliminary SAR and selectivity tests to guide in lead choice and optimization. The results on the new scaffold, based on a piperazine ring connected to a 1,3,5-triazine core, are indeed very promising. The optimised leads built from compound 7 represent good expectations in the quest for new drugs against schistosomiasis.

All the data are very well explained and all the methods are clearly presented, either in the manuscript or in the numerous supplementary files.

**Comment:** One curiosity on the bioinformatics tools/software used: it is true that many programmes nowadays are available to perform both homology modelling and ligand docking, but which is the point to give only one alternative in the manuscript? I refer to the sentence at p.4 paragraph 3 "Modeller could be a valid alternative"; a similar sentence at p. 5 paragraphs 3 and 4 and in Figure 1 "AutoDock could alternatively be used .."

If Authors have used only one programme (MOE and Glide) for each purpose and for any good reason, why give only one alternative? There are many others out there.

**Response:** During this study, MOE and Schrödinger (including Glide) were used for the homology modelling and the *in silico* virtual screen, respectively. However, these are licence managed software packages and, upon request of Wellcome Open Research’s editorial team, we were encouraged to suggest at least one freely available alternative that readers/referees could use to replicate our study.

**Comment: **On the other hand, if the Authors have used both programmes (MOE and Modeller; Glide and AutoDock), then it could be wise to give a comparison of the results based on the two softwares.

**Response:** As mentioned above, only one software was used for each task so we can’t provide a comparison of results as requested.

**Comment: **The rationale of the research project is clear and the manuscript reads easily, without the need to go back and forth to the supplementary files, unless for an in depth examination and thorough understanding.

A few minor comments/suggestions:

**Comment:** In the keywords Authors might add MLL, unless there is a limit given in the journal's instructions;

**Response: **The selection of the keywords was done during the submission process and we are not sure if this can be changed now. If so, we’ll update the list with ‘MLL’ as keyword.

**Comment:** p. 4 column 1 line 1: there is a typo in  "ad libitum";

**Response:** We have made corrections as suggested.

**Comment: **p. 4 column 1 lines 7 and 11: the buffer used is PBS. 1x does not mean anything, unless you give the recipe

**Response:** Detailed description of buffer composition was included in the manuscript.

**Comment:** p. 4 column 1, paragraph 2 line 1: "double saline solution" instead of 2x saline;

**Response: **We have made corrections as suggested.

**Comment: **p. 4 column 1 "Homology modelling". Does 58% sequence similarity between human and schistosoma MLL refer to the SET domain alone? In that case, please use both PDB ID and chain ID (here 5F6K:C);

**Response: **We have made corrections as suggested.

    

**Comment: **p. 5 SBVS framework: why have the Authors used the OPLS_2005 force field instead of the newest OPLS3?

**Response: **The licence we have used only allowed us to apply the OPLS_2005 force field rather than the newest one.

**Comment: **Figure 1 panel C legend: “Same orientation as in B, but highlighting the structural motifs I to IV in black.” instead of “Schematic representation of Smp_138030’s domain architecture with indication of the different motifs (black regions on the protein represented as green ribbon)”.

**Response: **We have made corrections as suggested.

**Comment: **The yellow ribbon is “peptide form histone H3”, not “histone protein”.

**Response: **We replaced the expression “histone protein” with “peptide from histone H3” as a more appropriate description.

    

**Comment: **Figure 2 legend: a space is missing between “indicated.All siRNA”

**Response: **This is probably a layout-related issue. A space is correctly present in the manuscript.

    

**Comment: **Figure 2 vs Fig. 4,5,7: why the p-values of figure 2 are different from those of the other figures? Are the cut-off different? Moreover the ** value is missing in figure 2.

**Response: **The *p* values illustrated in Figure 2 are derived from the Student’s two tailed *t* test, unequal variance - Mann-Whitney U-test. On the other hand, a different test was used for the data presented in Figure 4, 5 and 7.  Here, a Kruskal-Wallis ANOVA followed by Dunn’s multiple comparisons test was used; hence, the statistical significance values are differently presented.

 ** value was included in the updated version of the manuscript.

    

**Comment: **Figure 4B: for clarity it would be better to present the data from 0 to 50 and then a broken y axis line and start again from 300 to 400, so that the tiny differences between PZQ and compound 7 could be highlighted.

**Response: **This is a good suggestion, however, there was complete inhibition of egg production after treatment with compound 7 (all concentrations tested except for 6.25 uM) and no eggs were recovered from the positive control (PZQ).

    

**Comment: **Figure 5 legend: were the measurements done in duplicate or in triplicate?

**Response: **As indicated in the Figure legend, Panel A and Panel B measurements were performed in duplicate.  Measurements in Panels C and D were conducted in duplicate during two independent screens (4 measurements in total).

    

**Comment: **Table 3: SI for compound 20 is >24, not ND

**Response: **Based on Reviewer#2’s comments, we have decided to remove the SI column from Table 3.

    

**Comment: **Figure 6: the title of this figure should be the same as that of Figure 5 with the exception of Set 3. The present title is misleading → it cites schistosomula and then presents the egg counts.

**Response: **This is a good suggestion. We have updated the title using the expression “Anti-schistosomal activity”, which should cover all the different results presented in the figure and to avoid repeating the same title of Figure 5.

    

**Comment: **Figure 6C and 6D vs 4B and 5D: why are the DMSO egg counts at 150 (6C,D) instead of 300 (4B,5D)?

**Response: **The egg count data for the negative control DMSO is different because the number of worm pairs used for the experiment was different. For Figures 4 and 5, three worm pairs were used for each condition, so the egg counts in the DMSO control are in the range of 300 eggs. For Figure 6, only one pair worm was used so the DMSO control has fewer eggs (~150). Figure 4, 5 and 6 legends were updated accordingly. The methodology was updated as well.

    

**Comment: **Figure 7: p-values are missing in the legend.

**Response: **To avoid repetition, we refer to Figure 4 for the statistical analysis information as the same approach was used (Kruskal-Wallis ANOVA followed by Dunn’s multiple comparisons test) and the p-values are as indicated in Figure 4’s legend. However, as suggested, the figure legend was updated to incorporate the specific *p* values accordingly.

    

**Comment: **p. 13 column 2 “Discussion”: please use “Therefore” instead of “Because of this realisation”

**Response: **We have made corrections as suggested

**Comment: **p. 14 column 2 line 4: insert a “,” between “maintenance” and “including”

**Response: **We have made corrections as suggested 

We would like to thank the Reviewer and the Editorial Team very much for their detailed and very constructive comments, which have helped us to improve our manuscript. We hope that this point-by-point response could clarify the open questions and that our suggested revisions are clear and convincing. We are, of course, happy to discuss any further open issues.

Kind regards,

Gilda Padalino (on behalf of the authors)
